# Functionalization of MWCNTs for Bioelectrocatalysis by Bacterial Two-Domain Laccase from *Catenuloplanes japonicus*

**DOI:** 10.3390/nano13233019

**Published:** 2023-11-25

**Authors:** Azat Abdullatypov, Pavel Oskin, Veronika Fedina, Liubov Trubitsina, Sofiya Yakimovich, Ekaterina Shuvalova, Pradeep Verma, Tatyana Dyachkova, Olga Ponamoreva, Sergey Alferov

**Affiliations:** 1 Biotechnology Department, Tula State University, Pr. Lenina 92, 300012 Tula, Russia; azatik888@yandex.ru (A.A.);; 2 Institute of Basic Biological Problems RAS—A Separate Subdivision of Federal Research Centre Puschino Scientific Center for Biological Research RAS, Institutskaya Str., 2, 142290 Pushchino, Russia; 3 Laboratory of Ecological and Medical Biotechnology, Tula State University, Friedrich Engels Street 157, 300012 Tula, Russia; pavelfraj@yandex.ru (P.O.); agapovaweronica@yandex.ru (V.F.); olgaponamoreva@mail.ru (O.P.); 4 Institute of Biochemistry and Physiology of Microorganisms RAS—A Separate Subdivision of Federal Research Centre Puschino Scientific Center for Biological Research RAS, Prospekt Nauki, 5, 142290 Pushchino, Russia; 5 Department of Microbiology, Central University of Rajasthan Bandar Sindri, 613 E Haribhau Upadhaya Nagar, Ajmer 305817, India; 6 Department of Technology and Methods of Nanoproducts Manufacturing, Tambov State Technical University, 106/5, Building 2, Sovetskaya Str., 392000 Tambov, Russia

**Keywords:** carbon nanotubes, MWCNTs, laccase, *Catenuloplanes japonicus*, electrochemical measurements

## Abstract

This study was carried out in order to assess several modifications of carbon nanotube-based nanomaterials for their applications in laccase electrodes and model biofuel cells. The modified MWCNTs served as adapters for the immobilization of laccase from *Catenuloplanes japonicus* VKM Ac-875 on the surface of electrodes made of graphite rods and graphite paste. The electrochemical properties of the electrodes were tested in linear and cyclic voltammetrical measurements for the determination of the redox potential of the enzyme and achievable current densities. The redox potential of the enzyme was above 500 mV versus NHE, while the highest current densities reached hundreds of µA/cm^2^. Model biofuel cells on the base of the laccase cathodes had maximal power values from 0.4 to 2 µW. The possibility of practical application of such BFCs was discussed.

## 1. Introduction

One of the most important reactions for the wide use of fuel cells is the oxygen reduction reaction. This reaction is the limiting stage of electrocatalysis in hydrogen-oxygen proton exchange fuel cells, and a major part of noble metal catalysts is used on the cathodic part of the fuel cells. This limits the wide use of fuel cells because of the scarcity of platinum group metals on the Earth [1]. In order to overcome such limitations, the research is carried out in several directions: reduction of the amount of platinum per current unit [1,2], and the invention and optimization of platinum-free oxygen reduction catalysts [3,4], including biological catalysts like various oxidases [5].

To increase the current densities of oxygen electrodes, modification of the surface with carbon nanotubes is widely applied. Carbon nanotubes allow the achievement of multifold expansion of the real electrochemically active surface and reduce amounts of catalysts used per unit area of oxygen electrode [6]. The possibility of surface functionalization of nanotubes by attaching the desired side groups like phenolic [7], aryl [8,9,10], and heterocyclic groups [11], allows for the immobilization of various catalysts [12,13].

One of the possibilities for using the active surface-expanding property of carbon nanotubes in fuel cell technology is attaching enzymes. Enzymes, such as hydrogenases and oxidases, are considered renewable catalysts for the future of the fuel cell industry. For example, some parameters such as the specific activity of the catalyst per mole are better in hydrogenase than in noble metals [14]. Oxidases, such as laccases, are also more active than noble metals when considering the specific molar activity [15]. Moreover, there are previous works showing that the laccase of *Trametes versicolor* outperforms Pt catalysts by the overpotential value required for the onset of the oxygen reduction reaction [15,16]. To date, however, there is no vehicle running on enzymatic fuel cells, because the current densities of modern hydrogenase-oxidase fuel cells are at least one order of magnitude lower than the values required for the proper functioning of high energy-consuming machines [17,18,19]. This is due to certain disadvantages of biocatalysts, such as low stability and inhibition by a number of substances. However, their benefits include a high catalytic efficacy, the ability to function in mild conditions, the capability of oriented immobilization on the electrode surface, and they are caused by the protein nature of the enzymes. The search for enzymes of better properties and the design of biointerfaces, including the application of nanomaterials for proper orientation of protein catalysts, are the actual tasks of the research aiming to create competitive enzymatic biofuel cells (EBFCs). EBFCs are regarded as future batteries for net zero carbon emission energy [20], and also as power supplies for implanted biomedical devices [21].

The functioning of enzymatic electrodes is based on the phenomenon of bioelectrocatalysis. Bioelectrocatalysis is the coupling of biocatalytic reactions involving redox enzymes with electrode reactions (heterogeneous electron transfer). Direct-electron-transfer (DET)-type bioelectrocatalysis involves the direct interaction between enzymes and an electrode. DET-type bioelectrocatalysis imparts enzymatic characteristics to the electrode, creating simple systems with low energy losses [22]. In 1978, an important event was reported as a DET-type bioelectrocatalysis for the first time; catalytic reduction of O_2_ by a multi-copper oxidase (MCO) laccase (Lac) adsorbed on carbon electrodes [23].

Laccases (EC: 1.10.3.2, para-diphenol oxidases) are universal phenol-oxidizing enzymes capable of four-electron reduction of oxygen to water. These copper enzymes belong to the “blue oxidase family”. Their active site is composed of four copper atoms coordinated by imidazole groups of histidine residues [24,25,26]. The ability of laccases to transfer electrons to oxygen to form water is widely used in bioelectrocatalysis processes aimed at manufacturing oxygen electrodes for various applications including fuel cells [13,27,28].

Currently, laccase is assigned to the group of multicopper oxidases, a group including various proteins with different substrate specificity and various biological functions (copper efflux oxidase involved in copper homeostasis, bilirubin oxidase, ceruloplasmins (ferroxidases), ascorbate oxidases, and nitrite reductases) [29]. Based on the quantity of cupredoxin-like domains, multicopper oxidizes are divided into two-domain enzymes (2d), three-domain enzymes (3d), and six-domain enzymes (6d).

An example of 6d enzymes is ceruloplasmin, the main copper-containing protein of mammalian blood plasma with a function of oxidizing Fe^2+^ to Fe^3+^ by dissolved oxygen [30]. Its high redox potential value is very attractive from the point of view of developing an oxygen reduction catalyst functioning at minimal overpotential values (the property currently held only by platinum catalysts), but the electrochemical studies of this protein revealed that it is really hard to achieve an oxygen reduction current after its immobilization onto electrode surfaces. Only recently, Aleksejeva and colleagues achieved the first measurable oxygen reduction currents with ceruloplasmin cathodes, but the current density was only 1.65 µA/cm^2^ without a redox mediator and 12 µA/cm^2^ with a redox mediator [31].

Structurally all the multicopper oxidases contain at least four copper atoms, which are designated differently. Based on their spectroscopic features, copper sites can be divided into three categories reflecting the electronic and geometric structure of the active site: type 1 (T1) or blue copper, type 2 (T2) or normal copper, and type 3 (T3) or coupled binuclear copper centers [32]. Most laccases found in fungi, plants, and insects, contain three cupredoxin domains (T1, T2, and T3), and they are able to function in the monomeric form [33,34,35].

The catalytic activity of MCOs is dependent on the redox potential of the T1 copper center which is responsible for intermolecular electron transfer from substrates to the enzyme followed by intramolecular electron transfer from the T1 center to the trinuclear T2/T3 cluster. Usually, the redox potential of the MCOs lies in the range from 400 to 800 mV vs. NHE, and numerous factors affect the exact value of the redox potential of these enzymes [13]. Commonly, bacterial laccase has a lower redox potential of the T1 center than fungal enzymes. However, bacterial enzymes possess some advantages, such as higher thermal stability, the ability to function in a broad range of pH, the ability to oxidize phenolic compounds in alkaline conditions, and tolerance to inhibition by halides and azides. The majority of bacterial laccases are represented by two-domain enzymes with one T1 domain and one T2/T3 domain containing mixed-type copper ions. They are mostly active in homotrimeric form where three T1 atoms are located rather close to each other and connect the copper atoms of the trimer into the united electron transfer system [36,37,38,39]. A recent search of effective dye-decomposing enzymes among bacterial laccases found two-domain laccases with a relatively high redox potential of a T1 center [40] Laccase from *Catenuloplanes japonicus*, VKM Ac-875, which was characterized by Trubitsina and colleagues. It demonstrated the ability to oxidize various azo dyes and triarylmethane dyes. The initial attempt to measure its redox potential using ferrocyaninde-ferricyanide redox pair did not result in accurate values, which meant that the actual redox potential of the enzyme was either below 310 mV or above 460 mV [40]. However, its ability to decolorize dyes meant that its potential is at least above 400 mV, so the actual redox potential values should be 460 mV or higher.

**Figure 1:**
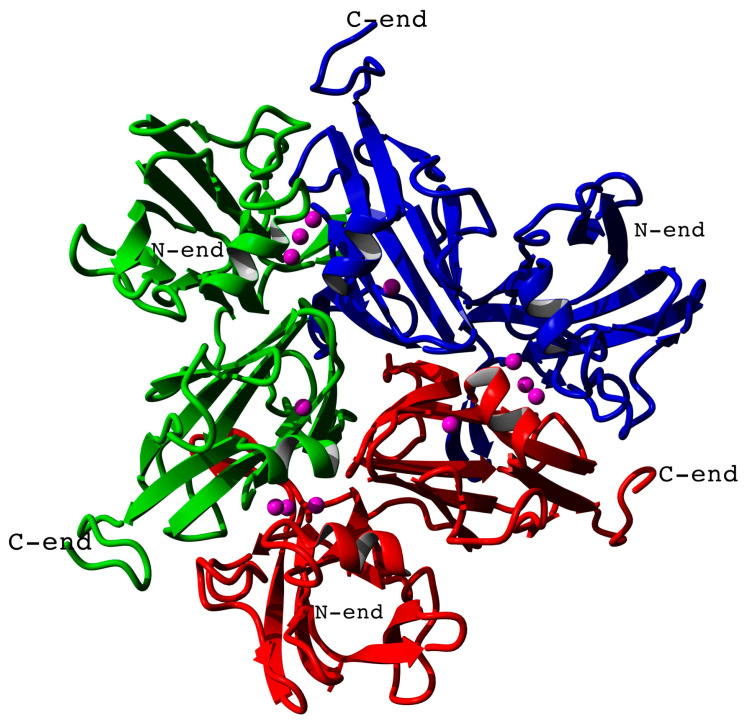
Homology model of the three-dimensional structure of *Catenuloplanes japonicas* multicopper oxidase (laccase). Separate chains are colored red, green, and blue. Copper atoms are shown as magenta spheres. N- and C-termini of the chains are labeled.

The model of the three-dimensional structure of *Catenuloplanes japonicas* laccase in its homotrimeric form (Figure 1) was previously demonstrated in [40]. The enzyme has three T1 copper centers in proximity to the center of the trimer, while T2/T3 copper atoms are located on the periphery.

An important aspect of achieving the direct electron transfer from the electrode to the T1 center of the enzyme is the proper orientation of the protein. In the current study, several types of modified carbon nanotubes were used to immobilize Ac-875 laccase onto the carbon electrode surface. It should be noted that the nanomaterials we used were the MWCNTs from a single batch, which is important for comparative analyses. The main idea of the present study was to test the feasibility of the application of MWCNTs functionalized by several different types of surface groups, including substrate-mimicking naphthyl groups, for immobilization of bacterial laccase from *Catenuloplanes japonicas* VKM Ac-875, and to assess its electrochemical properties as an oxygen-reducing biocatalyst, particularly, in biofuel cell (BFC) cathode compartment. The bioanode comprised a graphite electrode with immobilized natural enzymatic cascades from the membrane fraction of acetogenic bacterium *Gluconobacter oxydans* coupled to MWCNTs in hydrogel [41].

## 2. Materials and Methods

### 2.1. Enzyme Isolation

The enzyme used in the study was the bacterial two-domain laccase from *Catenuloplanes japonicus*, VKM Ac-875, expressed in *E. coli* and isolated as described earlier [40]. Briefly, the gene of the enzyme was cloned into a pQE-based vector without a TAT translocation signal in frame with a hexahistidine tag (His-tag), then the induction of expression was performed by adding IPTG, and the protein was solubilized, purified on a Ni-NTA column and dialyzed in Tris-HCl buffer with a pH of 9.0 for long-term storage.

### 2.2. Multiwall Carbon Nanotubes Processing and Measurements

Multiwall carbon nanotubes “Taunit-M” (Nanotechcenter LLC, Tambov, Russia) were produced by CVD synthesis from a propane-butane mixture on a Co/Mo catalyst. According to the manufacturer’s data, their external diameter is 10–30 nm, internal diameter is 5–15 nm, length ≥ 2 µm, and specific surface exceeds 270 m^2^/g (determined by BET method).

### 2.3. Oxidation of MWCNTs

MWCNTs (10 h of oxidation and 5 h of oxidation) were obtained in accordance with the work [42] by treating MWCNT “Taunit-M” with chemically pure boiling nitric acid for 10 and 5 h, respectively. After oxidation, MWCNT was separated from excess acid, and washed on a filter until a neutral pH flow was reached, and the resulting aqueous dispersion of the product was dried using freeze drying in a Scientz-10N freeze dryer. MWCNT oxidized by hydrogen peroxide vapors was obtained by treating “Taunit-M” MWCNTs in vapors of a 35% aqueous solution of hydrogen peroxide for 10 h at 120 °C [42].

### 2.4. Naphthylation of MWCNTs

**Figure 2:**
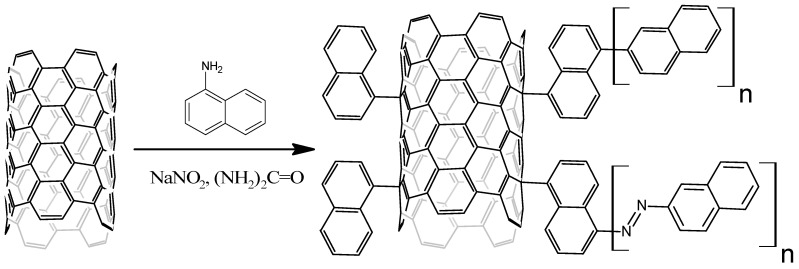
Functionalization of MWCNTs by naphthyl groups.

Functionalization of MWCNTs by naphthyl groups was performed by their treatment with arene diazonium salts in molten urea according to Figure 2 (NB: to make Figure 2 clearer, only one-half of the MWCNT cylinder is shown).

A total of 50 mg of MWCNTs were supplied with melted urea and constant stirring at 140–150 °C, and the mixture was homogenized for 5 min. Then, 200 mg of α-naphthylamine was added and mixed for 5 min. After α-naphthylamine supplementation, 276 mg of solid sodium nitrite powder was added, and the evolution of gaseous nitrogen was observed. The obtained reaction mixture was stirred for 5 min and poured into an Erlenmeyer flask with 200 mL of distilled water. The resulting suspension was filtered through a PTFE (0.22 μm) membrane filter. The ready naphthylated MWCNTs were washed several times with hot distilled water, then with DMSO, and acetone dried in the vacuum of a water jet pump.

### 2.5. Characteristics of MWCNTs

Granulometric composition and zeta potential were determined by dynamic light scattering using the NICOMP 308-ZLS instrument (NICOMP, Urbana, IL, USA). The structure of surface graphene layers was investigated by X-ray analysis using an ARL Equinox 1000 diffractometer. The IR spectra were obtained on FTIR spectrometer JascoFT/IR 6700 (JASCO UK Limited, W. Yorkshire, UK) in 500–4500 cm^−1^ range with 4 cm^−1^ step value. Combination scattering spectra were obtained on DXR Raman Microscope (Thermo Fisher Scientific, Waltham, MA, USA) at 532 nm excitation wavelength. Thermogravimetry/differential scanning calorimetry (TG/DSC) analysis was performed on synchronous thermal analyzer STA 449 F3 Jupiter (NETZSCH-Gerätebau GmbH, Selb, Germany) in air atmosphere in 40–900 °C temperature range, the heating rate was 10 °C/min. The number of acidic groups was determined by Boehm potentiometric titration [43,44] using Mettler Toledo Easy Plus (Mettler Toledo, Greifensee, Switzerland) automatic titrator.

### 2.6. Modification of Graphite Rod Electrodes (GRE)

The next step was to modify the surfaces of graphite rod electrodes with MWCNTs. MWCNTs were dispersed in deionized water with ultrasonic treatment. Due to the different wettability of the materials, naphthylated MWCNTs were taken in 5 mg per 10 mL of water, and ultrasonication was performed for 1 h. The concentration of oxidized MWCNTs was four-fold higher, 10 mg per 5 mL of water, and 10 min of ultrasonic treatment was enough to homogenize the dispersion. The graphite rod (Bruno Visconti Graphix (Markham, ON, Canada), HB, d = 2mm, S = 3.14 × 10^−2^ cm^2^) or spectral graphite rod (Research, Design and Technological Institute of Electrocoal Products, Electrougli, Russia, d = 8 mm, S = 3.52 cm^2^) was then immersed in the solution and dried in an oven at 100 °C for 1.5 h. The procedure was repeated four times. To ensure that only the top of the graphite rod was electrochemically active, the sides of the electrodes were coated with dielectric lacquer.

### 2.7. Modification of Carbon Paste Electrode (CPE)

The electrodes were prepared according to the following technique. A syringe was filled with graphite paste-mineral oil mixture (100 mg of graphite powder (Fluka, Darmstadt, Germany) per 40 µL of mineral oil (Fluka, Germany)). To modify CPEs with MWCNT-10, the modified multiwall nanotubes oxidized for 10 h were dispersed in deionized water (10 mg of MWCNT per 5 mL of water) by ultrasonication for 10 min. The prepared MWCNT-10 was applied to working electrodes (10 µL per electrode) and dried at room temperature.

Modification of CPE with MWCNT-H_2_O_2_ was as follows: 10 mg of MWCNT-H_2_O_2_ were dispersed in 10 mL of deionized water, and DMFA (1:1) by ultrasonication for 40 min. The prepared MWCNT-H_2_O_2_ was applied to working electrodes (10 µL per electrode) and dried at room temperature.

The diameter of the syringe neck used in the study was 1 mm, so the electrode area was taken for 0.785 square millimeters or 7.85 × 10^−3^ cm^2^. The electric contact between the carbon paste and metal contact wire excluded electrochemical corrosion due to the presence of mineral oil.

### 2.8. Determination of Electrochemically Active Surface (EAS)

Graphite pencil core electrodes (d = 2 mm) were installed into a three-electrode system with a silver chloride reference electrode and platinum foil counter electrode. The cell was filled with 10 mL of 1 mM K_3_[Fe(CN)_6_] in 0.1 M KCl, and cyclic voltammograms were recorded in the −400 to 600 mV potential range for scanning rates from 25 to 500 mV/s with 25 mV/s steps.

Oxidation and reduction peaks (anodic and cathodic peaks, respectively) were recorded. Then, the registered current and voltage values were used for the calculations according to the Randels-Sevcik equation [45]. The rate constant of heterogeneous electron transfer was calculated using the Nicholson-Lavagnini method [46].

### 2.9. Laccase Immobilization onto the Electrode Surface

Immobilization of laccase on the graphite pencil core electrode (d = 2 mm) was achieved according to the following simple technique. The pencil core modified with nanotubes was immersed into 100 µL of laccase from *Catenuloplanes japonicus* VKM Ac-875 (0.5 mg/mL) and dried on air for 1 h. Then the electrode was washed in sodium acetate buffer solution (pH 5.0).

Immobilization of laccase on the spectral graphite rod electrode (d = 8 mm) was carried out similarly. The nanotube-modified electrode was placed in 500 μL of *Catenuloplanes japonicus* VKM As-875 laccase solution (0.5 mg/mL).

Immobilization of laccase on the surface of graphite paste electrodes was performed by an encapsulation technique where 15 µL aliquot of enzyme solution (0.5 mg/mL) was applied to the modified CPE and dried at room temperature. The dialysis membrane (molecular mass cutoff of 4 kDa) was fixed on the electrode surface with a plastic/rubber ring and left for 12 h in sodium-acetate buffer solution (pH 5.0) at room temperature.

### 2.10. Amperometric Measurements

The measurements were performed by means of the electrochemical system Corrtest CS Studio (“CorrTest Instruments”, Wuhan, China). This appliance allowed us to perform measurements in both two-electrode and three-electrode schemes. In a two-electrode scheme, the electrodes (working electrode and Ag/AgCl reference electrode) were immersed into an electrochemical cell containing 10 mL of Na acetate buffer solution (pH 5.0). In a three-electrode scheme, an additional counter electrode made of Pt plate (S = 6 cm^2^) was added. Time dependence of the current was measured at constant potentials (0, 50, 100, 150, 200, 250, 300 mV, and higher until the value at which the direct electron transfer current reaches zero). First, the cell was purged with argon in order to remove oxygen, then, when a constant current was achieved, the buffer solution was saturated with oxygen by turning on the magnetic stirrer and shutting the argon line off.

Another approach was realized as follows. When both the argon line and magnetic stirrer were off, initial currents were measured. After the stabilization of the current, argon saturation was started. When the current grew higher and reached the anaerobic plateau, the argon line was shut, and the magnetic stirrer was switched on to saturate the cell with oxygen. The currents shifted to more negative values and reached another plateau. The potential at which both the initial current and aerobic plateaus were negative, and the argon plateau was positive, was considered as redox potential of the enzyme.

To compare the laccase currents with the catalysts used in fuel cells, graphite paste electrodes with the platinum catalyst PM20 (Prometey, Rostov-on-Don, Russia) were used. It is a nanosized carbon-based catalyst with an average particle size around 2.5 nm and a 20% mass fraction of platinum. The electrochemically active surface of this catalyst is around 80 m^2^ per g of platinum [47]. To produce the PM20 electrodes, the catalyst was applied to graphite paste electrodes instead of laccase solution (20 mg per electrode). The character and potential dependence of oxygen reduction currents were compared with laccase electrodes (see the Section 3).

### 2.11. Spectral Graphite Rod Electrodes and Biofuel Cell Model with Gluconobacter oxydans Natural Enzyme Cascades

To perform the test of *Catenuloplanes japonicus* laccase in the fuel cells, model biofuel cells (BFCs) were constructed, where the laccase served as a cathodic chamber catalyst.

Cultivation of *Gluconobacter oxydans* and obtaining bacterial natural enzyme cascades was performed according to the method described in [41]. In the anodic chamber, natural enzyme cascades of bacteria *Gluconobacter oxydans* were immobilized on the surface of an electrode and then fixed by conducting a matrix on the base of chitosan with MWCNT-5. A spectral graphite rod, d = 8 mm, modified with three varieties of carbon nanotubes (MWCNT-5, carbon nanotubes oxidized by hydrogen peroxide, and naphthylated carbon nanotubes) was used in the cathode chamber. As in previous experiments, the electrode was immersed in a solution of dispersed CNTs and then dried in an oven at 100 °C for 1.5 h, the procedure was repeated four times. Then the graphite rod was immersed in the solution of *Catenuloplanes japonicus* laccase and fixed on the surface with a BSA-based protein film.

### 2.12. Molecular Docking Studies

Coronene molecules modified with surface groups (carboxyl, lactone, hydroxyphenyl, and naphthyl) were built in the YASARA Structure and their energy was minimized using the YASARA NOVA force field [48]. Since these molecules served as models of carbon nanotube surface, they were built in two variants, wall-modified (modification at the center of the coronene ring) and edge-modified (modification at the edge of the ring). Then, they were transformed into PDBQT files in MGL-Tools [49]. Docking calculations were performed in Autodock VINA [50] when the docking box was centered at the center of mass of three T1-copper atoms, and the box size was set to 80 Å. The exhaustiveness value characterizing the completeness of the conformational search was set to 20, and the results of docking were analyzed in YASARA Structure. The distances from coronene planes to the closest copper atoms were measured.

## 3. Results

### 3.1. Characteristics of the Pristine and Modified Carbon Nanotubes

When determining the content of protogenic groups by the Boehm method, their content of CNTs oxidized in hydrogen peroxide vapors was below the sensitivity threshold of the method (˂0.1 mmol/g). Data on phenolic, lactone, and carboxylic groups in MWCNT-5 and MWCNT-10 are presented in Table 1. The content of protogenic groups differed insignificantly.

**Figure 3:**
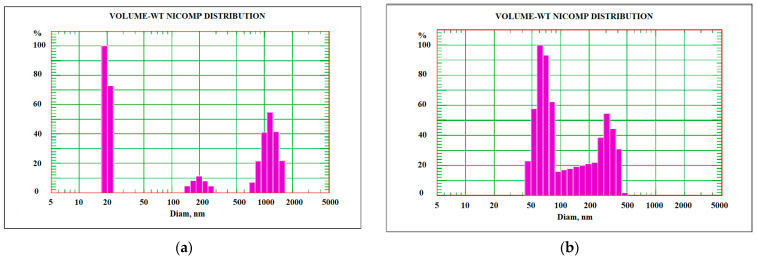
Granulometric composition of aqueous suspensions of pristine MWCNTs (**a**) and naphthylated MWCNTs (**b**) obtained by dynamic light scattering on NICOMP 308-ZLS.

When determining the granulometric composition, it was found that the dispersion of the pristine MWCNTs was composed of three main fractions with mean size values of 20 nm, 200 nm, and 1200 nm. After the modification, two fractions were observed with mean size values of 65 and 317 nm. Disintegration of large MWCNT aggregates (1200 nm) occurs due to a large quantity of the functional groups on the side surface of the nanotubes which impede the adhesion of the nanotubes to each other [51]. The disappearance of the 20 nm fraction is related to the increase in particle size via naphthalene polymerization [52]. In general, the size distribution became more uniform after naphthylation (Figure 3). The measurement of the oxidized nanotube aggregates did not show such a difference with the pristine nanomaterial [53].

Due to the polymodal dispersion of the MWCNT systems, the plot of zeta-potential measurement had two extremes. Both pristine and naphthylated MWCNTs were unstable in aqueous dispersions from the point of view of aggregation properties, because their zeta-potentials (22.62 and 23.31 mV, respectively) were below the threshold value (30 mV) [43] for the majority of the particles. A slightly higher zeta potential value for the naphthylated MWCNTs could also be explained by less aggregation propensity because of surface naphthyl functional groups.

**Figure 4:**
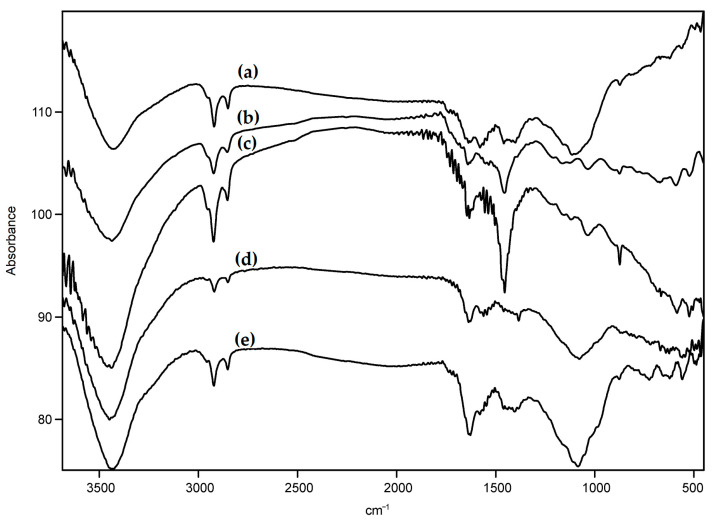
FTIR-spectra of the MWCNTs: (**a**) pristine MWCNTs “Taunit-M”, (**b**) MWCNTs oxidized in nitric acid for 5 h (MWCNT-5), (**c**) MWCNTs oxidized in nitrous acid for 10 h (MWCNT-10), (**d**) MWCNTs oxidized in hydrogen peroxide vapor (MWCNT-H_2_O_2_), and (**e**) naphthylated MWCNTs.

The vibration modes of O-H bonds (3400 cm^−1^), C=O bonds (1734 cm^−1^), C-O bonds (1200 cm^−1^), and C-H bonds (2800 and 2950 cm^−1^) are present in the IR spectra of raw and oxidized MWCNTs. (Figure 3). The oxygen-containing groups were present in the raw carbon materials due to the interaction of free carbon valences with oxygen from the air upon fabrication of the nanotubes [54]; their density increased upon oxidation (their intensity was compared using the vibration band of C-H bonds as an internal standard). The presence of methyl and methylene groups is a specific attribute of nanotubes obtained by the CVD method. The presence of absorption bands of naphthyl radicals in the spectra of naphthylated MWCNTs could hardly be detected due to their low intensity (Figure 4).

**Figure 5:**
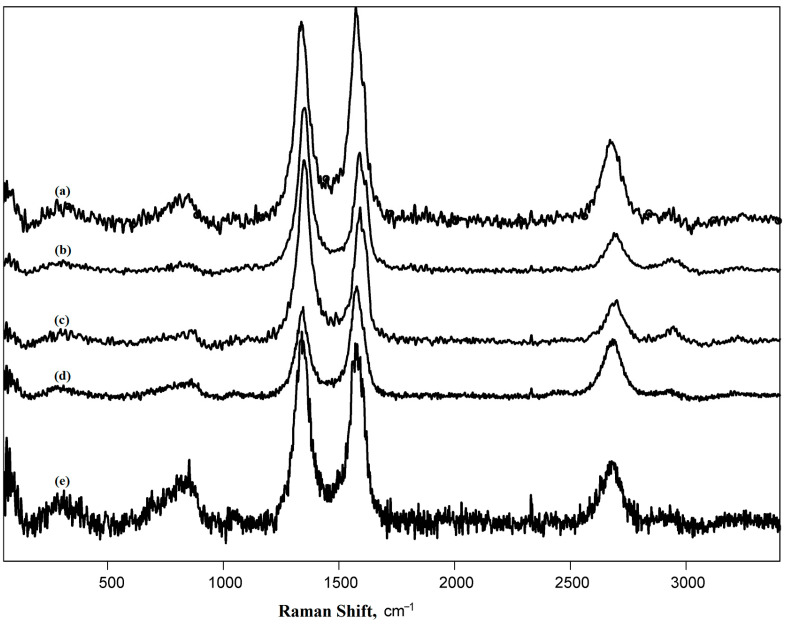
Raman spectra of MWCNTs: (**a**) pristine (raw) MWCNTs “Taunit-M”, (**b**) MWCNTs oxidized for 5 h in nitric acid (MWCNT-5), (**c**) MWCNTs oxidized for 10 h in nitric acid (MWCNT-10), (**d**) MWCNTs oxidized in hydrogen peroxide vapor (MWCNT-H_2_O_2_), and (**e**) naphthylated MWCNTs.

Raman spectroscopy is one of the most fruitful methods for studying carbon materials. It is not very time-consuming and provides abundant information on the surface structure [55], and the correlations of electrochemical properties and data from Raman spectra data are of great interest (Figure 5).

To interpret the absorbance bands, the Raman spectra of pristine and functionalized MWCNTs were processed according to the paper [56]. Then the density of the defects (**n_D_**) [57], the distance between the defects (**L_D_**) [57], and the size of crystallites (**L_a_**) were calculated [58]. The data of processed Raman spectra are summarized in Table 2.

The shape of the Raman spectrum was typical for other CNTs [55], and the data were in agreement with other studies on the same nanotubes [41,42]. The high intensity of the 2D band reflected a high degree of order of nanotube structure [59]. Functionalization of MWCNTs increased the count of defects, which is clear from the ratio of D and G bands, D′ and G bands, as well as from the values of defect density and inter-defect distances [55]. According to the ratio of D″ and G intensities, oxidation under mild conditions (hydrogen peroxide vapor and boiling in nitric acid for 5 h) led to the complete removal of the amorphous phase, whereas with 10 h of boiling in nitric acid, partial amorphization of the material occurred [60]. Naphthylation did not affect the degree of amorphization of CNTs. The type of graphite crystalline lattice defects can be revealed from the ratio of D and D’ bands [61,62]. In the pristine MWCNTs and MWCNTs oxidized under mild conditions, edge defects were more specific, while the functionalized MWCNTs were mostly characterized by local defects. The presence of D* in the spectrum of naphthylated CNTs gave evidence of the elevated content of sp^3^-hybridized carbon atoms, which confirmed the presence of substituting groups bound to the surface carbon atoms [60]. A comparison of the obtained data with the literature would not be correct due to the individual settings of each Raman spectrometer [63].

**Figure 6:**
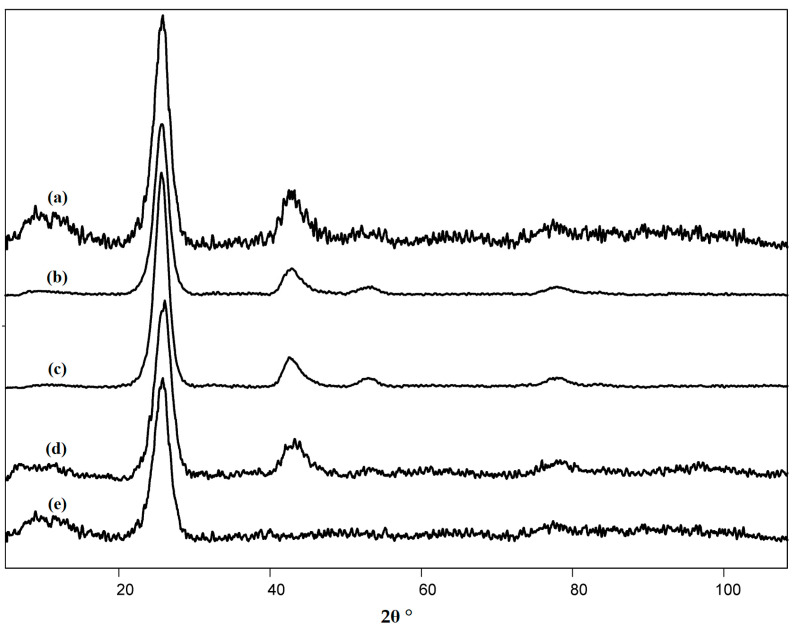
The diffractograms of MWCNTs used in the work: (**a**) pristine (raw) MWCNTs “Taunit-M”, (**b**) MWCNTs oxidized for 5 h in nitric acid (MWCNT-5), (**c**) MWCNTs oxidized for 10 h in nitric acid (MWCNT-10), (**d**) MWCNTs oxidized in hydrogen peroxide vapor (MWCNT-H_2_O_2_), and (**e**) naphthylated MWCNTs.

The pristine and naphthylated nanotubes were characterized by X-ray diffractometry. The diffractogram obtained for the pristine MWCNTs was recorded and described earlier [42], the sharpness of the peaks and the absence of curvature indicated a high structuring of the nanomaterial [42]. The diffractogram is shown in Figure 6.

The diffractogram had 2 symmetrical reflexes at 2θ, equal to 25.9 and 43.4°, with interplane distances of 3.4 and 2.9 Å. There was only one pronounced symmetrical reflex at 2θ = 26.3° on the diffractogram of naphthylated MWCNTs. This value is most close to 26.6°, which is specific for graphite [64]. In this case, d002 was 3.4 Å. In the process of naphthylation, the value of d002 did not change, hence no changes in the interlayer space of nanotubes occurred [65]. Most likely, the layer of poly-naphthalene on the surface of nanotubes was not large, which had a beneficial effect on their electrochemical properties. Nevertheless, after naphthylation, the 43.4° reflex disappeared, which indicated a decrease in the degree of crystallinity of the nanomaterial. This conclusion is consistent with the Raman spectroscopy data (Table 2), namely, with a slight increase in the intensity of peaks D” (amorphous phase) and 2D (number of graphene layers).

**Figure 7:**
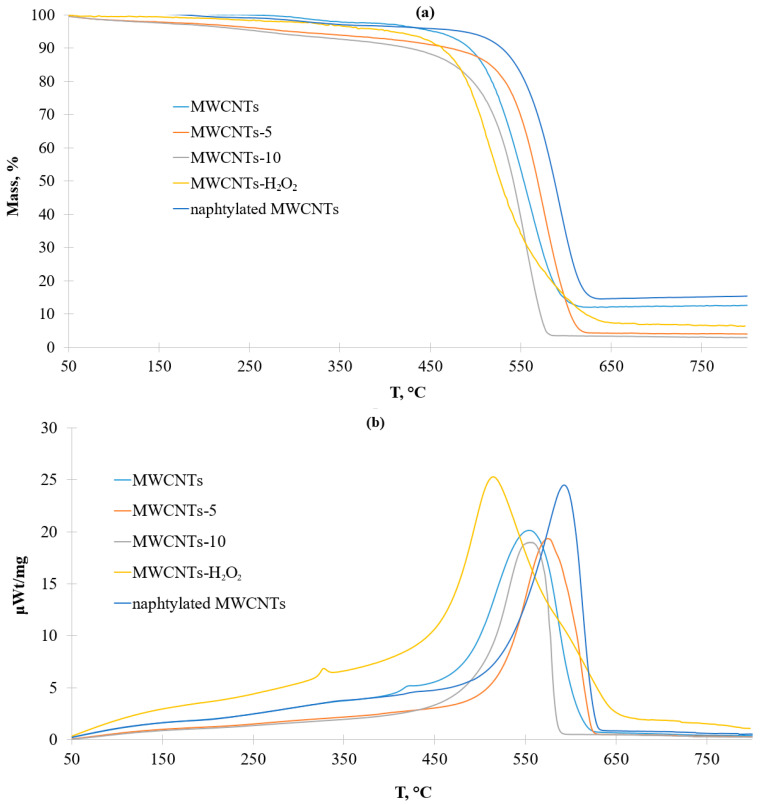
(**a**) TG-curves for different MWCNTs, and (**b**) DSC-curves for different MWCNTs.

To assess the degree of functionalization and thermal stability of naphthylated MWCNT, TG/DSC curves were obtained in the comparison of pristine MWCNT (Figure 7). Data for oxidized CNTs were described in the literature earlier [42,43].

According to the results of TG/DSC, it showed that naphthylated MWCNTs are more thermally stable than the initial ones (decomposition temperature was 560 °C at the initial and 600 °C with naphthylated ones). In addition, naphthylated MWCNTs on the DSC curve did not have a local maximum of 400 °C, corresponding to the removal of oxygen-containing groups.

The local maximum on the DSC curve of pristine MWCNTs at about 420 °C characterized the removal of the amorphous phase which is often present in CVD-derived carbon nanotubes [66]. According to the paper [67], the initial stages of MWCNTs oxidation involve the removal of amorphous carbon, which results in an observable decrease of ID/IG ratio and a change of synchronous thermal analysis results. Particularly, the DSC curves of MWCNT-H_2_O_2_ had a local maximum at 330 °C, not at 420 °C, This peak was specific for the removal of oxygen-containing groups [68]. Upon oxidation in concentrated nitric acid, the hydrophilicity of MWCNTs and the number of surface defects increased. The mass decrease of MWCNTs oxidized in HNO_3_ up to 250 °C was due to the removal of adsorbed water. Then, the destruction of surface functional groups occurred. Due to the higher functionalization degree of the MWCNTs oxidized by nitric acid for 10 h, their complete destruction occurred faster than in other MWCNTs used in this study. The naphthylated MWCNTs were more thermostable than the raw MWCNTs (the decomposition temperature of pristine MWCNTs was 560 °C, and that of naphthylated ones was 600 °C). Moreover, naphthylated MWCNTs did not possess the local maximum around 420 °C corresponding to the removal of the amorphous phase. The addition of sodium nitrite to molten urea apparently led to nitroso urea formation which is a diazo reagent. The interaction of nitrosourea with MWCNTs led to the decomposition of the amorphous phase.

The electrochemical behavior of functionalized MWCNTs was investigated by cyclic voltammetry (See Supplementary Figure S1) using potassium hexacyanoferrate (III) as an electrochemical sensor. The electrochemically active surface area (EAS), the rate constant of heterogeneous electron transfer (k_s_), and the fraction of the edge plane (f_e_) were determined (Table 3).

Modification of the graphite pencil core with functionalized MWCNTs led to an increase in EAS. This effect was least noticeable in the case of naphthylated MWCNTs, which may be due to the lower affinity of the hydrophilic ferricyanide for the hydrophobic naphthyl radicals. For oxidized MWCNTs, the EAS increased in the series MWCNTs-H_2_O_2_—MWCNTs-10—MWCNTs-5. This result can be explained by the following reasons. The oxidation in peroxide vapor introduces predominantly hydroxyl groups on the nanotube surface, which do not dissociate under the experimental conditions (pH of distilled water is around 5–6 units), which does not increase the wettability of the material significantly. The edge plane fraction of graphite is one of the most important characteristics of the electrochemical properties of carbon materials. The edge plane of graphite plays a key role in electron transfer in a number of electrochemical systems [69], making a major contribution to the formation of the capacitance of the electric double layer [70]. The catalytic properties of carbon nanomaterials with respect to oxygen reduction are also due to the high content of the edge plane [71]. For the investigated carbon materials, the fraction of the edge plane increased in the series: blank pencil rod—naphthylated MWCNTs—MWCNTs-5—MWCNTs-H_2_O_2_—MWCNTs-10. This series did not correlate with the density of defects on the surface of the material, which can be explained by the different wettability of MWCNTs with different functional groups.

### 3.2. Determination of Redox Potential of Ac-875 Laccase in Bioelectocatalytical Systems Based on Graphite Paste Electrodes

**Figure 8:**
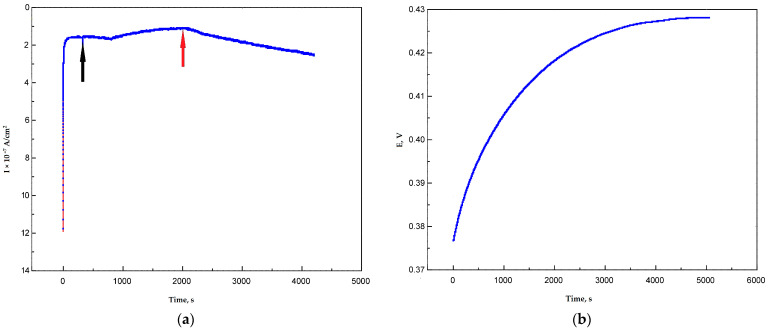
(**a**) Current of carbon paste-MWCNT-10 laccase electrode after purging the system with argon (black arrow) and restoration of oxygen supply (red arrow) at +300 mV against Ag/AgCl reference electrode. (**b**) OCP curve of carbon paste-MWCNT-10 electrode against Ag/AgCl reference electrode.

The following idea was used during the determination of the redox potential of Ac-875 laccase during the oxygen-argon-oxygen scheme. When the system was flushed with argon, the cathodic current (oxygen reduction reaction current) was close to zero. When the oxygen was provided via switching off the argon line and turning on the magnetic stirrer, the cathodic current appeared again, reflecting the prevailing of the main electrocatalytic process (reduction of oxygen) over the side electrocatalytic processes like oxidation of some surface groups by the laccase. In this methodology, if the applied potential was higher than the redox potential of the T1 center of the enzyme, the restoration of the cathodic current could occur, and the current line would be flat on the plateau. For this purpose, graphite paste electrodes were used (Figure 8).

It is worth noting that the difference between the electrodes modified with nanotubes with different surface functionalizations was insignificant, which can be explained by larger measurement errors in the range of smaller currents. At +300 mV vs. Ag/AgCl (+500 mV vs. NHE), the current values for the MWCNT-10 electrode were 0.001 µA, while for the MWCNT-H_2_O_2_ they were in 0.03–0.04 µA range. Taking into account the smaller area of carbon paste electrodes (7.85 × 10^−3^ cm^2^), the current density was equal to 0.127 µA/cm^2^ for MWCNT-10 and 3.822–5.096 µA/cm^2^ for peroxide-modified MWCNTs. At +250 mV vs. Ag/AgCl (+450 mV vs. NHE), the values were about 0.04 for MWCNT-10 and 0.06 for MWCNT-H_2_O_2_, which meant that the current densities were 5.096 µA/cm^2^ for the MWCNT-10 electrode and 7.644 µA/cm^2^ for MWCNT-H_2_O_2_. At +310 mV (+510 mV vs. NHE), there was only noise level current for all the nanomaterials tested. Therefore, the real redox potential values could be around +510 mV vs. NHE.

**Figure 9:**
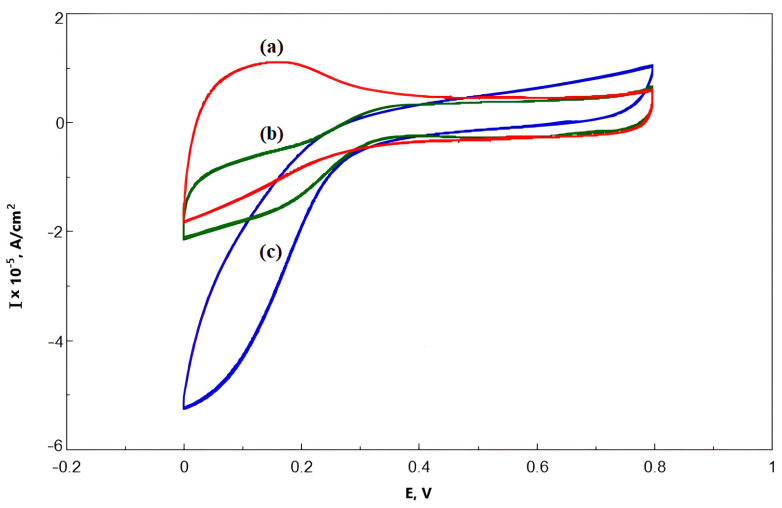
Cyclic voltammetry of MWCNTs-based laccase electrodes and PM20-loaded carbon paste electrode. (**a**) MWCNTs-10 and Ac-875 laccase, (**b**) MWCNTs-naphthyl and Ac-875 laccase, and (**c**) PM20-based electrode. The potential values are given against the Ag/AgCl electrode (0 mV corresponds to +200 mV vs. NHE).

Moreover, the open circuit potentials were measured for carbon paste electrodes, but the results were not stable enough. The OCP values of five tested electrodes were in the range from +400 to +460 mV vs. Ag/AgCl (+600–+660 mV vs. NHE), but the application of external potentials equal to those values resulted in positive currents meaning that some minor anodic process occurred at those conditions. When the same procedure was repeated with naphthylated MWCNTs, comparable results were achieved. At +300 mV (+500 mV vs. NHE) the electrode still reacted to the extra oxygen supply provided by switching the magnetic stirrer on. To compare the electrocatalytic properties of Ac-875 laccase immobilized on oxidized and naphthylated nanotubes with the commercial Pt-based catalyst PM20, cyclic voltammograms were recorded in a +800–0 mV potential range (+1000–+200 mV vs. NHE) at different scanning rate. Cyclic voltammograms for a 10 mV/s scanning rate are shown in Figure 9.

The shape of cyclic voltammograms for the laccase on naphthylated nanotubes looked more similar to the voltammogram for the PM20 catalyst. The anodic current observed on the voltammogram of the MWCNT10-Ac-875 laccase catalyst could reflect the process of oxidation of the surface groups of the nanotubes. Phenolic groups could be an example of oxidized groups. On the whole, the magnitude of laccase electrode current density and of current density of standard PM20 catalyst were of the same order, which means that the laccase-based electrodes could be used in bioelectrocatalysis as platinum analogs in some cases.

### 3.3. Comparative Analysis of Bioelectrocatalytic Oxygen Reduction on Graphite Pencil Rods Modified with Ac-875 Laccase Immobilized on Functionalized Carbon Nanotubes

The dependence of cathodic currents on the applied potentials was studied with all the functionalized nanotube types used in the work in the bioelectrocatalytical systems on the base of graphite pencil rods. They were modified with MWCNTs and Ac-875 laccase as described in Section 2.7 and Section 2.8. The pencil rod-based electrode attracts significant attention for various bioelectrochemical applications due to their high availability, low cost, simplicity of use, mechanical strength, chemical inertness, functioning in a wide potential range effective adsorption of electroactive substances, easiness of surface modification, low toxicity, and simple disposal procedure [72,73,74]. It should be noted that the surface of graphite pencil rod electrodes is renewable, it does not require strict polishing procedures, and it provides good reproducibility of the results [75].

**Figure 10:**
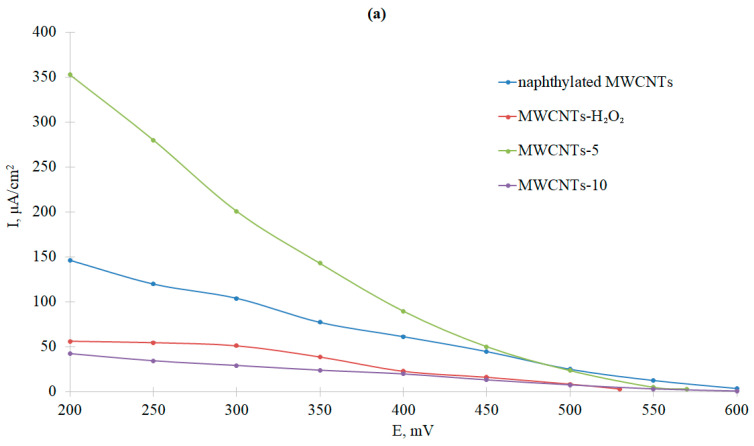

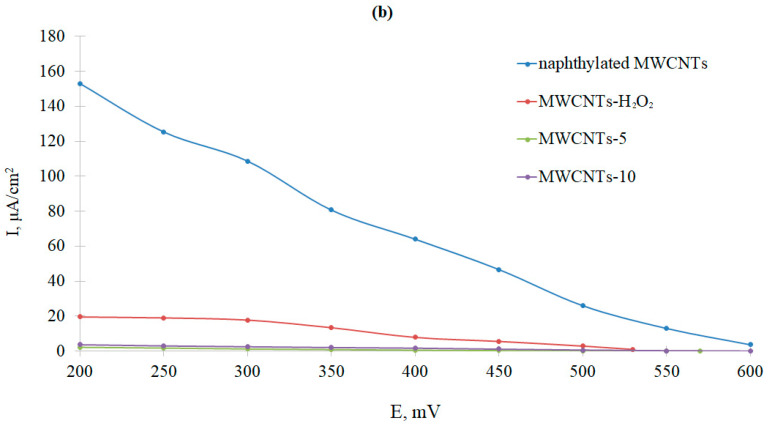
Dependences of current density on potential: (**a**) conversion to the visible (geometric) surface area, and (**b**) conversion to the electrochemically active surface area. Potentials are given as mV vs. NHE.

The current densities of bioelectrocatalytic currents were calculated using both visible and electrochemically active surfaces. EAS was calculated from the previous experiments with potassium ferricyanide (Table 3). The voltammograms are presented in Figure 10 (absolute values of cathodic currents depending on potentials vs. NHE).

The greatest oxygen reduction current was observed for nanotubes oxidized in nitric acid for 5 h (Figure 10a). Naphthylated nanotubes showed a slightly worse result in the reaction of bioelectrocatalytic oxygen reduction by the immobilized Ac-875 laccase. However, if we calculated the current density taking the electrochemically active electrode surface area, it was clear that the current density of direct electron transfer was an order of magnitude higher for naphthylated MWCNTs than for other variants. Thus, naphthylation (attachment of naphthyl substituting groups onto the electrode surface) contributed to the correct orientation of the laccase on the electrode surface. However, due to the high hydrophobicity of naphthyl groups, this type of functionalization did not lead to a significant increase in the electrochemically active surface area of the electrode. Therefore, in the future, it is necessary to combine the process of oxidation of MWCNTs with naphthylation, which will optimize the bioelectrocatalytic reduction of oxygen. The zero current potential, corresponding to the potential of the T1-center of laccase, was also determined. In all cases, it was approximately 500 mV vs. NHE, which was in accordance with the results from Section 3.2.

It should also be noted that the electrodes modified with MWCNTs-5 and naphthylated MWCNTs were able to reduce oxygen even in the absence of an enzyme. At 200 mV vs. NHE, the current densities in terms of the visible (geometric) surface area were 15.6 µA/cm^2^ and 0.38 µA/cm^2^, respectively (with laccase it was 350 and 150 µA/cm^2^, respectively). In the first case, this was due to the high content of the edge plane (Table 3), which played the role of a catalyst in oxygen reduction [71]. In the second case, it was due to the redox activity of the naphthalene/naphthoquinone system [76]. The lack of oxygen reduction on MWCNTs-10 was apparently due to their amorphization (Table 2).

### 3.4. Biofuel Cell with Anode Biocatalyst of Natural Enzyme Cascades of Bacteria Gluconobacter oxydans

First, optimal conditions for the modification of the anode were selected, and then the MWCNTs were compared with a different type of surface functionalization (Table 4). MWCNTs-10 were not used in cathode modification, as they performed worst in oxygen reduction experiments (Figure 10).

Thus, anode modification with MWCNT-5 led to a decrease in the energy parameters of the biofuel cell, since the anode biocatalyst was not properly fixed on the surface of the spectral graphite rod due to the large number of MWCNTs. However, the use of a chitosan-based matrix increased the energy parameters of the BFC, compared to the application of BSA protein film. This factor may be related to the instability of the BSA protein film, which came off the electrode surface during measurements and led to a decrease in the energy parameters of the BFC.

When modifying the cathode, the highest power (P) and the lowest internal resistance (R) were observed using naphthylated MWCNTs and MWCNTs-H_2_O_2_. The comparability of the parameters for BFC models could be explained by the fact that the anode process limits the power of the biofuel element. Since the modification of the anode led to shifts in current and power values, and power changes were not proportional to changes in OCP and closed potential, the cathode part definitely did not limit the energy parameters of the biofuel element. The closed-circuit potential for BFC models under study differed slightly, the open-circuit potential was maximal when modifying the cathode with MWCNTs-5, which may be due to their high surface area (Table 3).

### 3.5. Molecular Docking Results

Coronene, a polyaromatic hydrocarbon (PAH) composed of seven condensed aromatic rings and having D6h-type symmetry can be regarded as the elementary fragment of graphene [77]. The results of molecular docking of coronene derivatives mimicking the surface of MWCNTs used in the work were analyzed from the point of view of affinity and proper geometrical orientation of laccase on the electrode surface (Table 5).

**Figure 11:**
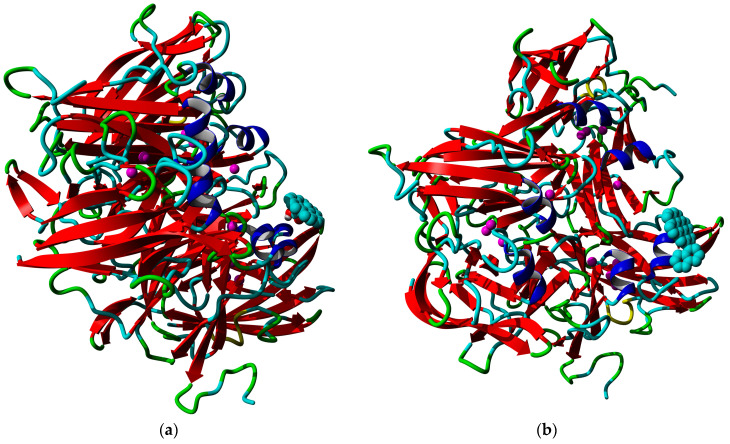
Realistic and non-realistic docking complexes of modified coronene derivatives and Ac-875 laccase: (**a**) realistic docking complex with a coronene molecule modified by a carboxyl group, and (**b**) non-realistic docking complex with a coronene molecule modified by naphthylation.

From the point of view of binding affinity (Gibbs energy of binding, the lower the stronger) and distance to copper (the shorter the more intensive electron transfer), it could be judged that the lactone-modified and naphthylated coronene molecules were the best substrates for interaction with laccase. However, not every docking complex reflected the situation occurring on the nanotube surface. The following Figure 11 shows an example of realistic and unrealistic docking complexes. The realistic complex means that it might take place when the enzyme is interacting with a nanotube, a part of which is represented by a modified coronene molecule. An unrealistic complex means that such interaction is impossible on the nanotubes (for example, when the modifying group would lie inside the nanotube in such a case, which is unrealistic from the physical point of view). The details of binding, as well as the formulae of all the coronene derivatives used in the work, are listed in Supplementary Figure S2.

From the point of view of the existence of realistic and non-realistic docking complexes, despite the center-modified carboxylated coronene having even a bit lower affinity towards the laccase than the unmodified coronene, it is quite a preferable anchoring agent for the laccase due to good distance towards the copper atom and the macroscopic parameters of the oxidized nanotubes such as high hydrophilicity. So, molecular docking is useful to interpret some of the peculiarities of laccase-MWCNT interaction, but some cases could be non-realistic due to restrictions on the size of molecules used. For example, we failed to prepare the same modifications using supercoronene as a starting point due to the limitation of the YASARA energy minimization tool.

## 4. Discussion

The application of nanomaterials in the immobilization of *Catenuloplanes japonicas* laccase led to current values comparable to those of platinum-based nanocatalysts. The values of current densities achieved were around hundreds of µA/cm^2^. One of the promising materials for bioelectrode development was buckypaper. A recent review provides a detailed analysis of the key parameters that determine the performance of buckypaper in energy conversion and storage applications, including biofuel cells [78]. Buckypaper modified with hemin was used to immobilize bilirubin oxidase [79]. The bioelectrode showed a direct electron transfer between multi-walled carbon nanotubes and bilirubin oxidase with an onset potential of 770 mV vs. RHE, but more efficient electron transfer was observed in the presence of the mediator 2,2-azino-bis(3-ethylbenzothiazoline-6sulfonic acid) (ABTS). This means that in order to increase the fraction of properly orientated enzyme molecules, it is necessary to continue research on the modification of carbon materials, which is initially easier to perform with MWCNTs. Comparative analysis of the current densities of bioelectrocatalytic oxygen reduction obtained in the work with modified MWCNTs allowed to reveal factors affecting this process, namely the orientation of the bacterial two-domain laccase near the electrode surface and change of the electroactive surface area of the electrode. The efficiency of direct electron transfer depends on the position and orientation of the enzyme [80], which is especially important for multicopper oxidases [81]. The T1-center of the enzyme should be oriented towards the electrode surface, and the preferred distance from the copper atom to the conductive fragment of the electrode should be less than 10 Å. [82]. Different strategies of oriented immobilization of MCOs have been developed, and other ones are still under development, which is summarized in one of the most recent reviews on laccase bioelectrocatalysis [13]. The mentioned strategies include the use of materials functionalized with ionogenic groups providing a positive or negative charge of the electrode surface [83], hydrophobic-hydrophilic interactions of protein molecules with nanomaterials [84], and modification of the electrode with nanomaterials with aromatic molecular linkers (substrate-mimicking compounds) [85,86,87,88]. Blanford et al. achieved effective direct (mediator-free) electron transfer via immobilization of fungal laccase onto pyrolytic graphite plane electrode after its modification with aryldiazonium. The modification of the carbon surface resulted in higher current density [85]. The same group used another amino derivative of polyaromatic compounds earlier for electrode modification by diazotization reaction. The electrocatalytic action of fungal laccase directly adsorbed onto the carbon electrodes is significantly improved after derivatization of the surface by polycyclic aromatic functional groups by diazonium conjugation of the corresponding amines. We used another method for MWCNT naphthylation including molten urea as a solvent. MWCNTs were perfectly dispersible in molten urea, which led to more complete functionalization of the surface. Moreover, there were no adverse reactions to naphthyl radical condensation, so the product was not contaminated by PAHs. Molten urea is also a “green” solvent, which makes our technique especially promising. We have demonstrated that bacterial two-domain laccase can adsorb onto the surface of naphthylated MWCNTs in an oriented manner and effectively participate in direct electron transfer.

Comparative analysis of bioelectrocatalysis efficiency on the base of type and modification degree of MWCNTs allowed to reveal critical aspects of MWCNT functionalization for fabrication of biocathodes on the base of bacterial two-domain laccase. The emergence of oxygen-containing groups on the surface of MWCNTs due to their oxidation by nitric acid for 5 h leads to elevated activity of the electrochemical system with laccase in more degree than naphthylation (Figure 8a). However, the naphthyl groups on the MWCNT surface provide proper orientation of the adsorbed enzyme, which facilitates effective direct electron transfer (Figure 8b). Oxidation in mild conditions led to preferential oxidation of the edges of MWCNTs, which did not provide the desired effect in bioelectrocatalysis. Prolonged oxidation of MWCNTs with nitric acid (for 10 h) is not recommended due to the formation of debris on the surface and amorphization of the carbon nanotubes. Debris decreases the electron-transfer capacity of MWCNTs [89]. Thus, the oxidation of MWCNTs and their naphthylation should be combined in future studies, allowing optimization of the process of bioelectrocatalytic oxygen reduction. When developing the flexible electrodes based on buckypaper, the results of experiments with modified MWCNTs and laccase should be taken into account.

The application of the Ac-875 laccase-MWCNTs as the cathode of a biofuel cell was carried out. The power per square centimeter reached 2 µW/cm^2^ for the best case of the biofuel cell with *Gluconobacter* natural enzyme cascades. Comparing this to the data described in the literature, it was still low for practical application. For example, the up-to-date record of current density for enzymatic oxygen reduction in hydrogen-oxygen fuel cells is 20 mA/cm^2^ [17]. In a direct methanol fuel cell with *Rhus vernicifera* laccase, it reached even higher values, 50 mA/cm^2^ [90].

The prospects of practical applications of biofuel cells based on bacterial laccases seem to be promising. At the present level of current and power densities, the biofuel cells with bacterial laccase anodes could be used for implanted medical devices fed with dissolved glucose and oxygen. While the anodic part of the BFC described in the work is probably very immunogenic, the cathodic part should at least be less immunogenic than fungal laccases or bilirubin oxidases. Our proposal is based on the presence of surface oligosaccharide moieties on the molecules of fungal enzymes. These moieties seem to be the most important reasons for the immunogenicity of the laccases from *Trametes versicolor* and bilirubin oxidase from *Myrrhothecium verrucaria*. From the point of view of immunogenicity, the ideal implanted BFC could be based on a ceruloplasmin cathode. However, the current density obtained with ceruloplasmin cathodes without a mediator was only 1.65 µA/cm^2^ [31]. Thus, the bacterial laccase could be an optimal variant possessing moderate current density values and (possibly) moderate immunogenicity compared to fungal enzymes. Besides the concern of immunogenicity, Ac-875 is quite tolerant to halogenide ions, whereas fungal laccases and bilirubin oxidases are more sensitive to them, including chloride ion that is the most common component of human blood salts.

Another approach in the development of further studies should be the elevation of redox potential values and current densities. The experiments on increasing the redox potential of laccase often result in a dramatic decrease in enzymatic activity, and this is definitely not the case for practical applications other than some sorts of potentiometric biosensing devices.

## 5. Conclusions

Thus, modified carbon nanotubes enabled us to obtain oxygen reduction currents from the immobilized laccase of *Catenuloplanes japonicus*. Recombinant laccase from *Catenuloplanes japonicus*, VKM Ac-875, is a relatively high-potential enzyme (redox potential above +500 mV vs. NHE), which makes it a promising cathode catalyst for manufacturing enzymatic and mixed-type fuel cells with various anode catalysts. Comparison of laccase-MWCNT catalysts with the commercial Pt-based catalyst showed that the current densities are of one order of magnitude, which means that the studied laccase could become a base for a promising Pt-substituting catalyst.

The achieved values of interaction affinity of laccase with carbon nanotubes or graphene layers can be to some extent modeled by molecular docking of correspondingly modified polycyclic aromatic hydrocarbons such as coronene.

## Figures and Tables

**Table 1:** Content of phenolic, lactone, and carboxylic groups in the oxidized MWCNTs according to titrimetric analysis.

Sample	Degree of Functionalization by the Groups (mmol/g)			
	-OH (Phenolic)	Lactone	-COOH (Carboxylic)	Total-Content
MWCNT-5	0.8	0.5	0.4	1.7
MWCNT-10	0.7	0.6	0.4	1.7

**Table 2:** Characterization of Raman spectra of pristine, oxidized, and naphthylated carbon nanotubes.

CNT Type	I_D*_/I_G_	I_D_/I_G_	I_D”_/I_G_	I_D′_/I_G_	I_D_/I_D′_	I_2D_/I_G_	L_D_, nm	n_D_ × 10^−9^, cm^−2^	L_a_, nm
Pristine MWCNT	-	1.27	0.11	0.38	3.34	0.35	10.7	273	13.0
Naphthylated MWCNT	0.02	1.53	0.12	0.74	2.06	0.45	9.8	330	10.8
MWCNT-H_2_O_2_	-	1.11	-	0.36	3.08	0.52	11.5	239	12.8
MWCNT-5	-	1.40	-	0.44	3.18	0.31	10.2	301	11.5
MWCNT-10	-	1.42	0.21	0.84	1.69	0.31	10.1	306	11.7

**Table 3:** Electrochemical characterization of pencil core electrodes modified with functionalized MWCNTs.

Type of Electrode	EAS, cm^2^	k_s_ × 10^4^, cm·c^−1^	f_e_, %
Blank pencil core electrode	0.011 ± 0.002	4 ± 1	0.4 ± 0.1
Electrode with MWCNTs-5	5 ± 1	150 ± 20	15 ± 2
Electrode with MWCNTs-10	1.2 ± 0.3	800 ± 300	80 ± 30
Electrode with MWCNTs-H_2_O_2_	0.09 ± 0.4	250 ± 80	25 ± 8
Electrode with naphthylated MWCNTs	0.03 ± 0.01	20 ± 0.4	2.00 ± 0.04

**Table 4:** Parameters of biofuel elements with various modifications of the anode and cathode.

**Modification of the Anode, the Cathode Was Modified by MWCNTs-5**				
**Type of Anode**	OCP, mV	CCP, mV	P, μW	R, kΩ
**Graphite rod +** **Chitosan + MWCNTs-5**	215 ± 9	115 ± 5	1.64 ± 0.02	9.5
**Modified graphite rod +** **Chitosan + MWCNTs-5**	125 ± 6	125 ± 3	1.4 ± 0.2	13
**Modified graphite rod + BSA + MWCNTs-5**	100 ± 7	120 ± 4	1.3 ± 0.1	13
**Modification of the laccase cathode, the anode was formed according to paragraph 2.11**				
**CNT type**	OCP, mV	CCP, mV	P, μW	R, kΩ
**Naphthylated MWCNTs**	120 ± 5	160 ± 3	5.3 ± 0.8	5
**MWCNTs-H_2_O_2_**	130 ± 4	145 ± 5	5.4 ± 0.9	4
**MWCNTs-5**	330 ± 17	135 ± 12	2.0 ± 0.1	10

**Table 5:** Results of molecular docking of coronene derivatives to the Ac-875 laccase.

Derivative	Binding Affinity, kcal/mol	Distance to Copper, Å
Coronene, unmodified	−7.9	11.464
Coronene, naphthylated (center)	−8.6	11.526
Coronene, naphthylated (edge)	−9.0	11.973
Coronene, phenolic (center)	−7.7	11.703
Coronene, phenolic (edge)	−8.4	12.577
Coronene, carboxylated (center)	−7.8	11.443
Coronene, carboxylated (edge)	−8.3	11.332
Coronene, lactone (center)	−8.6	12.274
Coronene, lactone (edge)	−8.8	11.600

## Data Availability

The [5] data relevant to the study could be provided by the corresponding author.

## Supplementary Materials

The following supporting information can be downloaded at: https://www.mdpi.com/article/10.3390/nano13233019/s1. Figure S1-1. CVs for initial pencil core electrode in the solution of 1 mM K_3_[Fe(CN)_6_] in 0.1 M KCl. Each colour corresponds to a different scanning rate ranging from 25 to 500 mV/s with an interval of 25 mV/s. Figure S1-2. CVs for pencil core electrode modified with MWCNT-5 in the solution of 1 mM K_3_[Fe(CN)_6_] in 0.1 M KCl. Each colour corresponds to a different scanning rate ranging from 25 to 500 mV/s with an interval of 25 mV/s. Figure S1-3. CVs for pencil core electrode modified with MWCNT-10 in the solution of 1 mM K_3_[Fe(CN)_6_] in 0.1 M KCl. Each colour corresponds to a different scanning rate ranging from 25 to 500 mV/s with an interval of 25 mV/s. Figure S1-4. CVs for pencil core electrode modified with MWCNT-H_2_O_2_ in the solution of 1 mM K_3_[Fe(CN)_6_] in 0.1 M KCl. Each colour corresponds to a different scanning rate ranging from 25 to 500 mV/s with an interval of 25 mV/s. Figure S1-5. CVs for pencil core electrode modified with Naphthylated MWCNT in the solution of 1 mM K_3_[Fe(CN)_6_] in 0.1 M KCl. Each colour corresponds to a different scanning rate ranging from 25 to 500 mV/s with an interval of 25 mV/s. Figure S2-1. Coronene molecule built in YASARA Structure. Atoms: cyan, carbon; gray, hydrogen. Bonds: gray, bond of order 1; magenta, bond of order 1.33; red, bond of order 1.5; orange, bond of order 1.67 yellow, bond of order 2. Figure S2-2. Coronene molecule carboxylated at the central position. Atoms: cyan, carbon; gray, hydrogen; red, oxygen. Bonds: gray, bond of order 1; magenta, bond of order 1.33; red, bond of order 1.5; orange, bond of order 1.67 yellow, bond of order 2. Figure S2-3. Coronene molecule carboxylated at the edge position. Atoms: cyan, carbon; gray, hydrogen; red, oxygen. Bonds: gray, bond of order 1; magenta, bond of order 1.33; red, bond of order 1.5; orange, bond of order 1.67 yellow, bond of order 2. Figure S2-4. Coronene molecule with lactone group at the central position. Atoms: cyan, carbon; gray, hydrogen; red, oxygen. Bonds: gray, bond of order 1; magenta, bond of order 1.33; red, bond of order 1.5; orange, bond of order 1.67 yellow, bond of order 2. Figure S2-5. Coronene molecule with lactone group at the edge position. Atoms: cyan, carbon; gray, hydrogen; red, oxygen. Bonds: gray, bond of order 1; magenta, bond of order 1.33; red, bond of order 1.5; orange, bond of order 1.67 yellow, bond of order 2. Figure S2-6. Coronene molecule with naphthyl group at the central position. Atoms: cyan, carbon; gray, hydrogen. Bonds: gray, bond of order 1; magenta, bond of order 1.33; red, bond of order 1.5; orange, bond of order 1.67 yellow, bond of order 2. Figure S2-7. Coronene molecule with naphthyl group at the edge position. Atoms: cyan, carbon; gray, hydrogen. Bonds: gray, bond of order 1; magenta, bond of order 1.33; red, bond of order 1.5; orange, bond of order 1.67 yellow, bond of order 2. Figure S2-8. Coronene molecule with phenol group at the central position. Atoms: cyan, carbon; gray, hydrogen; red, oxygen. Bonds: gray, bond of order 1; magenta, bond of order 1.33; red, bond of order 1.5; orange, bond of order 1.67 yellow, bond of order 2. Figure S2-9. Coronene molecule with phenol group at the edge central position. Atoms: cyan, carbon; gray, hydrogen; red, oxygen. Bonds: gray, bond of order 1; magenta, bond of order 1.33; red, bond of order 1.5; orange, bond of order 1.67 yellow, bond of order 2. Figure S2-10. Binding of coronene with central naphthyl substitution to Ac-875 laccase. Surrounding hydrophobic amino acids are labeled; green arrow shows the shortest distance to the T1 copper atom (11.517 Å). Figure S2-11. Binding of coronene with the edge phenol group to Ac-875 laccase. Hydrogen bonding network is shown as dashed lines; possible hydrogen bond between phenolic group and Arg399 is shown as solid yellow arrow; green arrow shows the shortest distance to the T1 copper atom (12.577 Å).
