# Supplementary material for: Functionalization of MWCNTs for Bioelectrocatalysis by Bacterial Two-Domain Laccase from Catenuloplanes japonicus
Source: Nanomaterials (Basel). 2023 Nov 25;13(23):3019. doi: 10.3390/nano13233019 (PMC10707984; doi:10.3390/nano13233019)
Supplement: Supplementary file 1 [file nanomaterials-13-03019-s001.zip › Supplementary Figure S1-1--5.pdf]

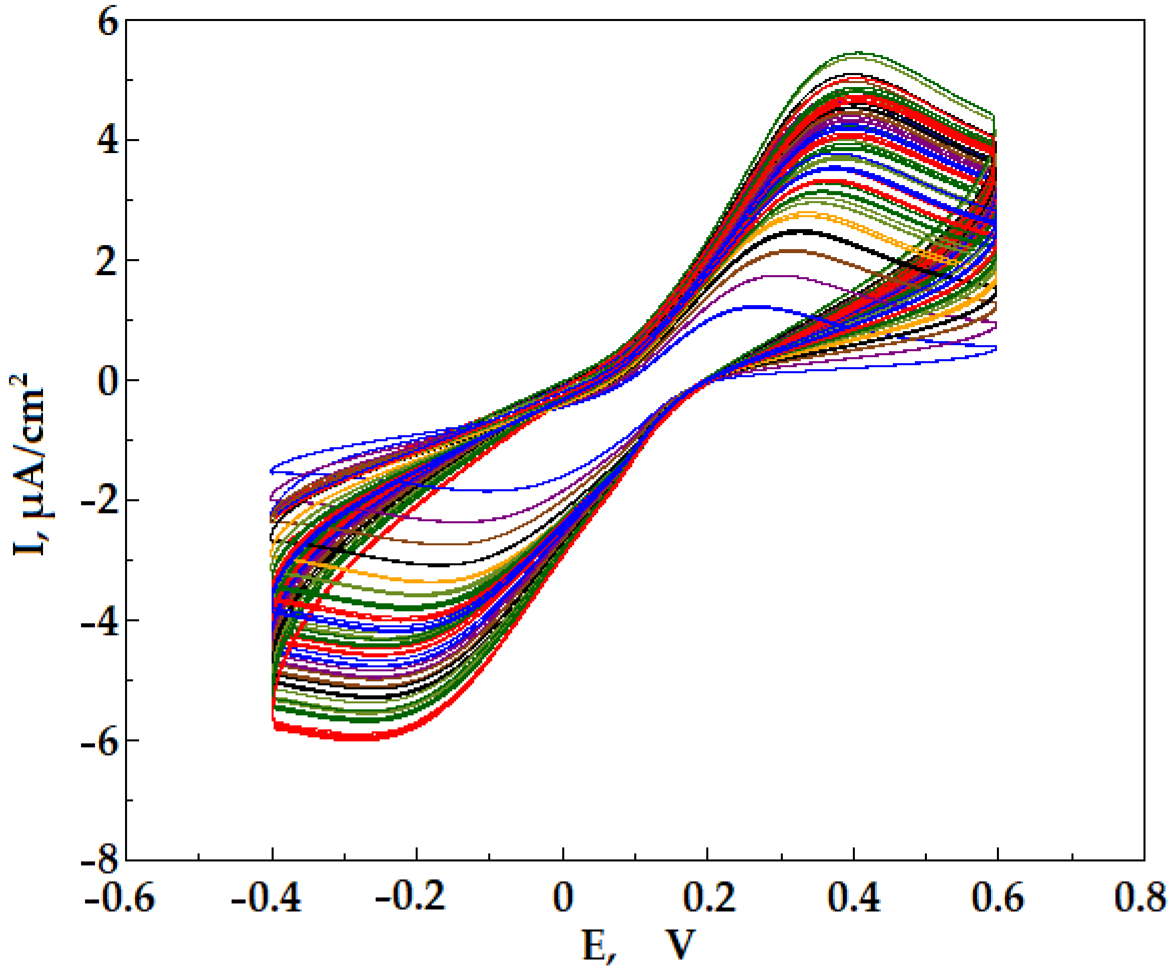

Figure S1-1. CVs for initial pencil core electrode in the solution of 1 mM $K_3[Fe(CN)_6]$ in 0.1 M KCl. Each colour corresponds to a different scanning rate ranging from 25 to 500 mV/s with an interval of 25 mV/s.

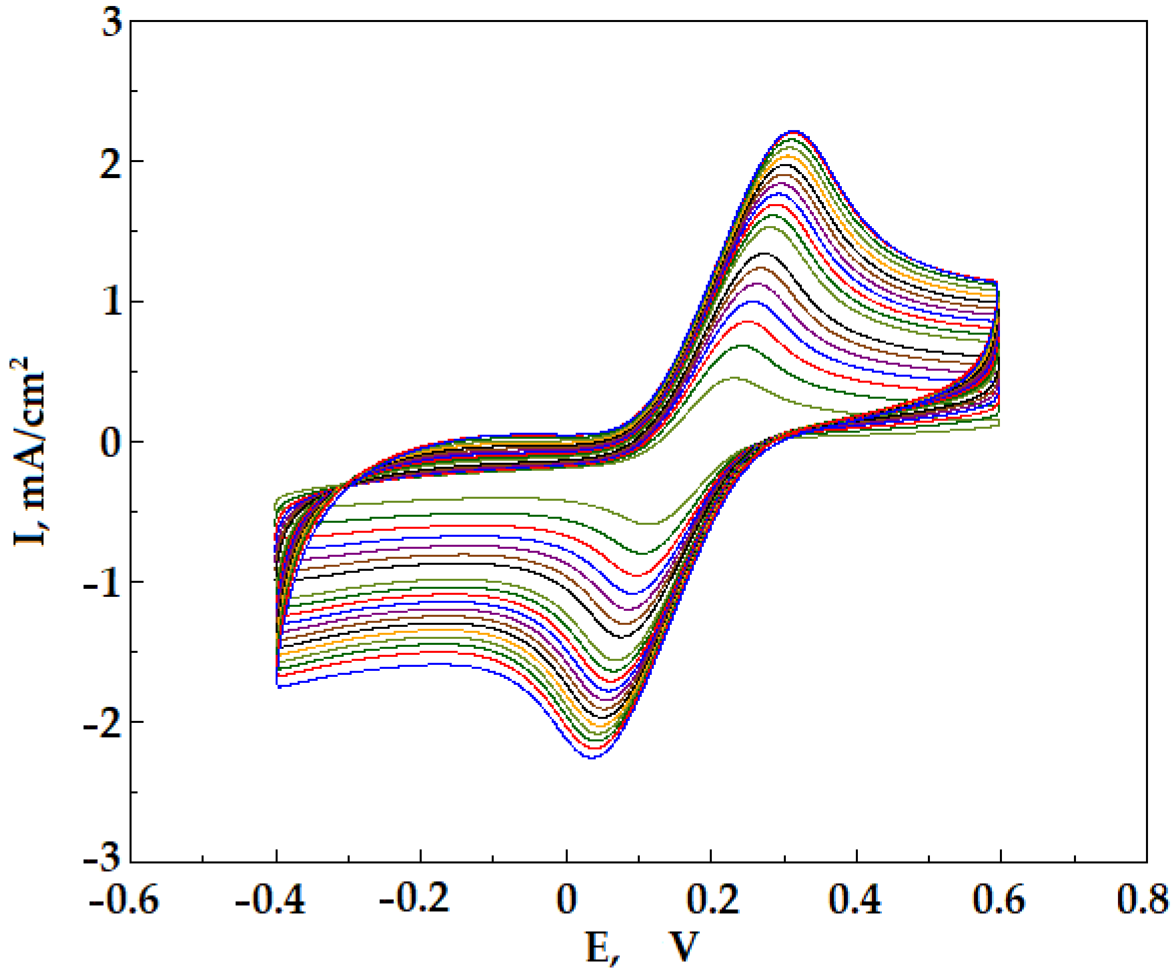

Figure S1-2. CVs for pencil core electrode modified with MWCNT-5 in the solution of 1 mM $K_3[Fe(CN)_6]$ in 0.1 M KCl. Each colour corresponds to a different scanning rate ranging from 25 to 500 mV/s with an interval of 25 mV/s.

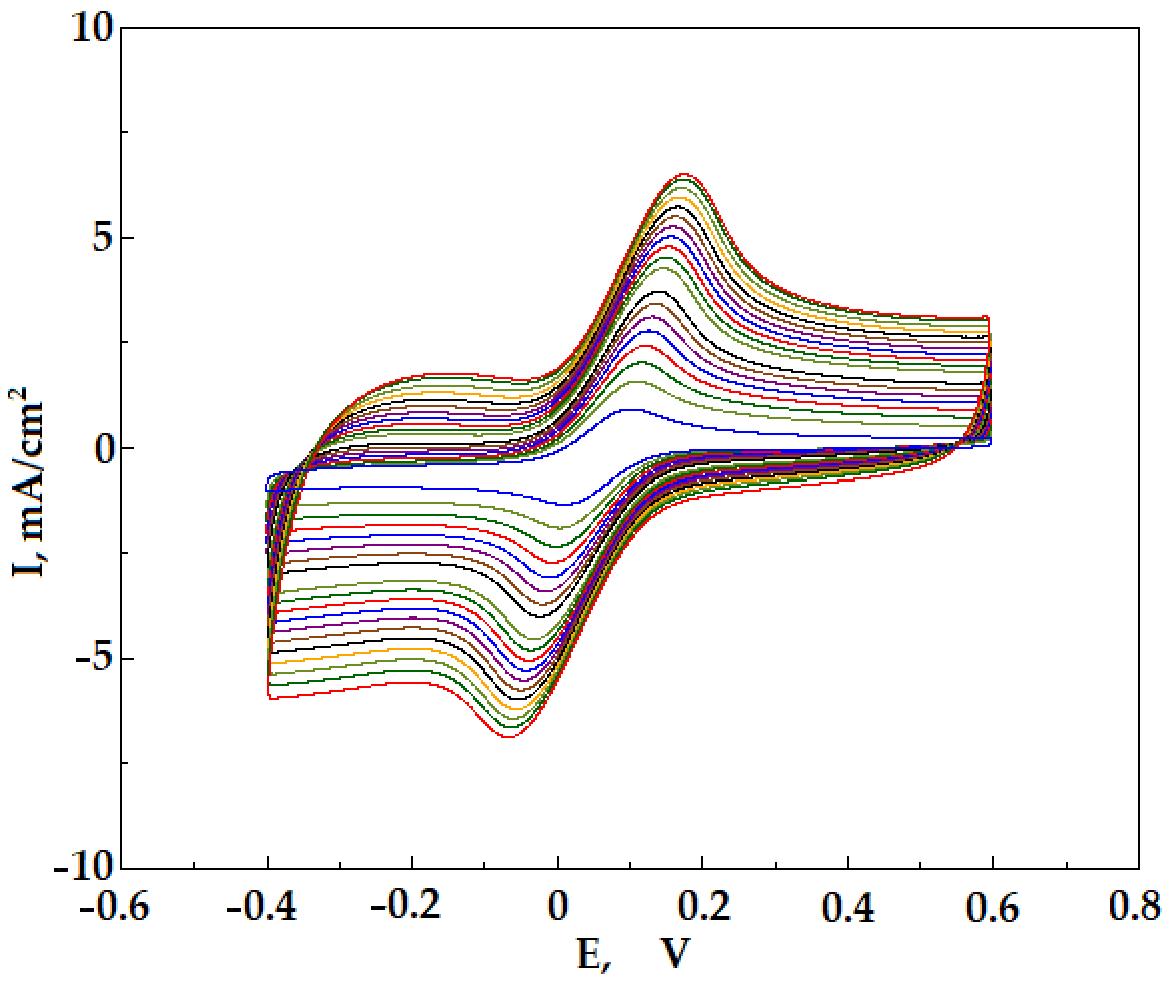

Figure S1-3. CVs for pencil core electrode modified with MWCNT-10 in the solution of 1 mM $K_3[Fe(CN)_6]$ in 0.1 M KCl. Each colour corresponds to a different scanning rate ranging from 25 to 500 mV/s with an interval of 25 mV/s.

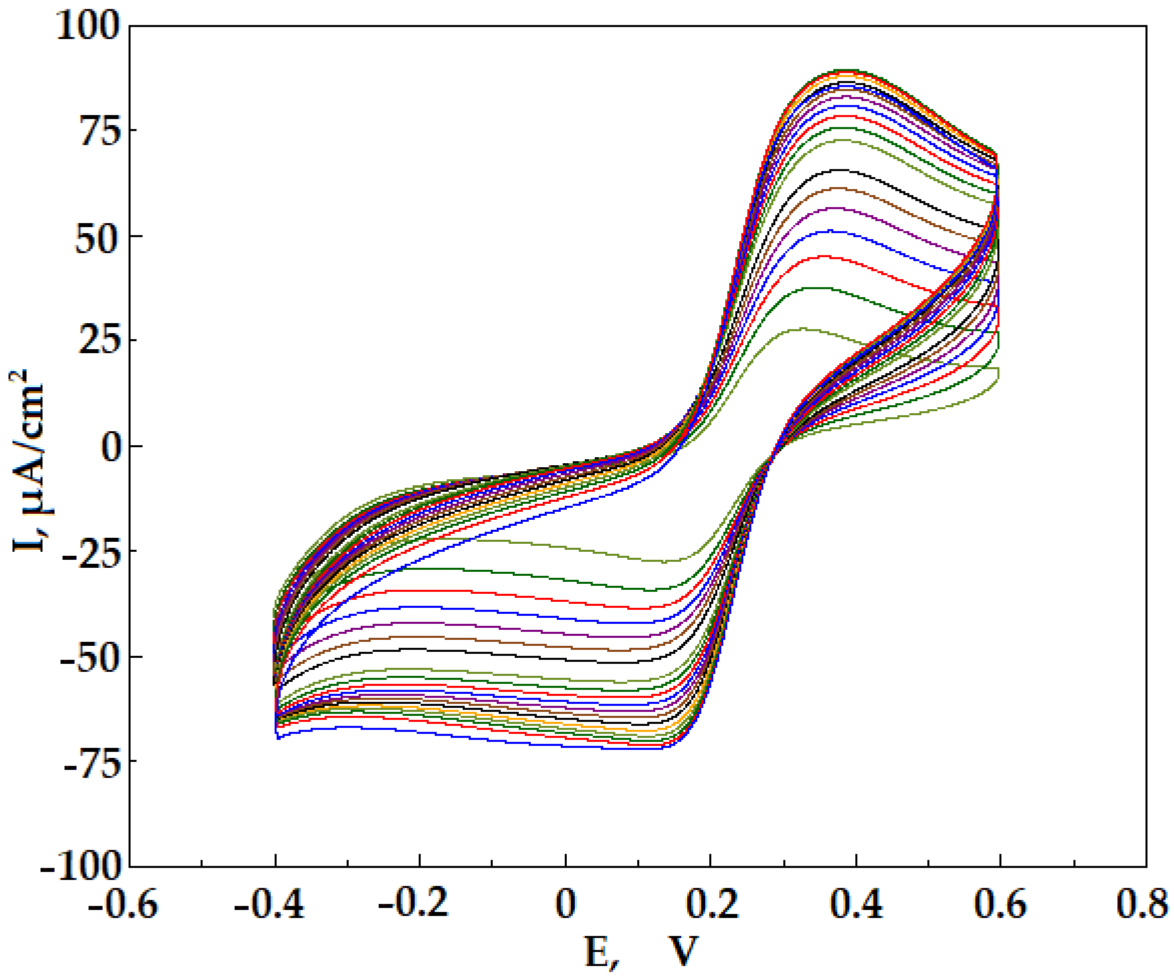

Figure S1-4. CVs for pencil core electrode modified with MWCNT-H_2O_2 in the solution of 1 mM $K_3[Fe(CN)_6]$ in 0.1 M KCl. Each colour corresponds to a different scanning rate ranging from 25 to 500 mV/s with an interval of 25 mV/s.

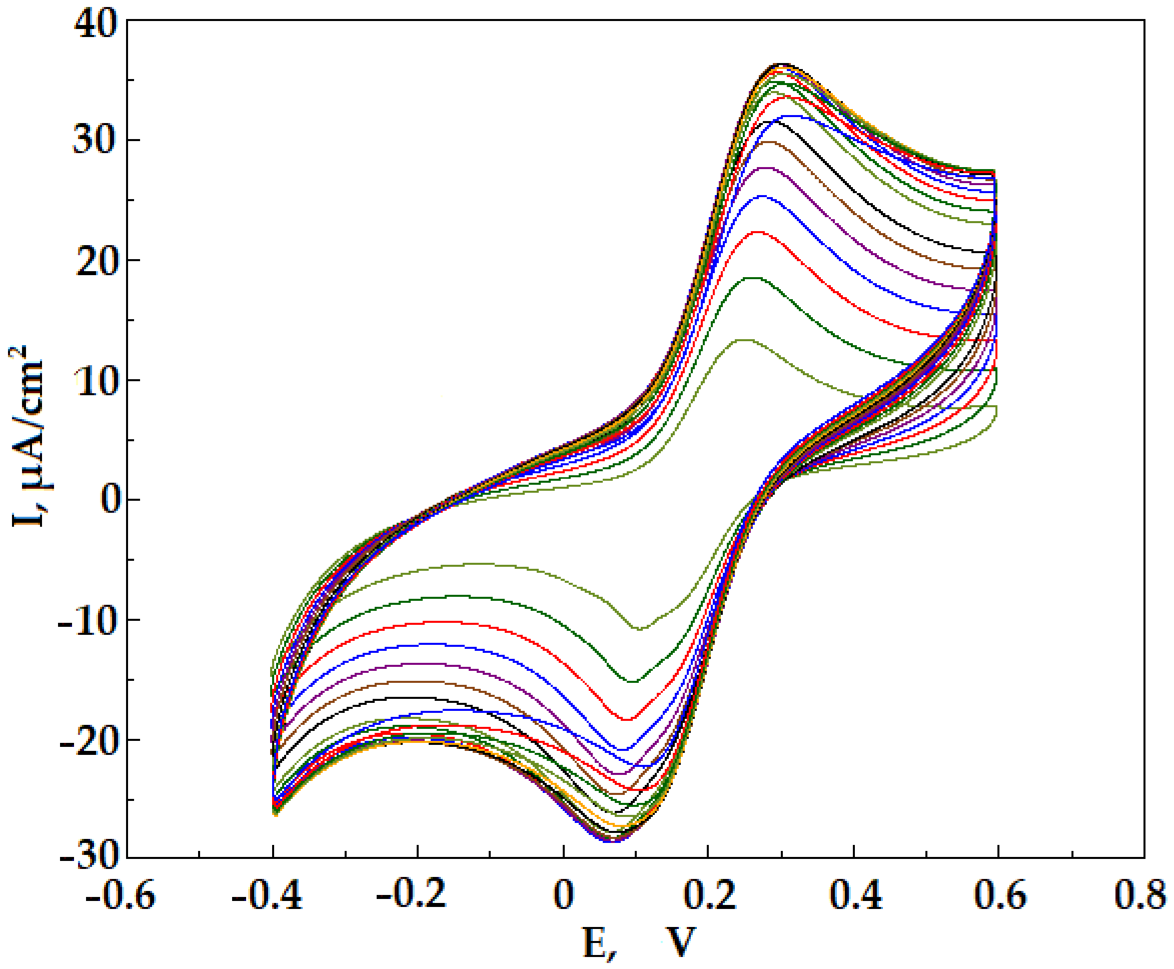

Figure S1-5. CVs for pencil core electrode modified with Naphthylated MWCNT in the solution of 1 mM $K_3[Fe(CN)_6]$ in 0.1 M KCl. Each colour corresponds to a different scanning rate ranging from 25 to 500 mV/s with an interval of 25 mV/s.
