# Supplementary material for: Functionalization of MWCNTs for Bioelectrocatalysis by Bacterial Two-Domain Laccase from Catenuloplanes japonicus
Source: Nanomaterials (Basel). 2023 Nov 25;13(23):3019. doi: 10.3390/nano13233019 (PMC10707984; doi:10.3390/nano13233019)
Supplement: Supplementary file 1 [file nanomaterials-13-03019-s001.zip › Supplementary Figure S2-1--11.pdf]

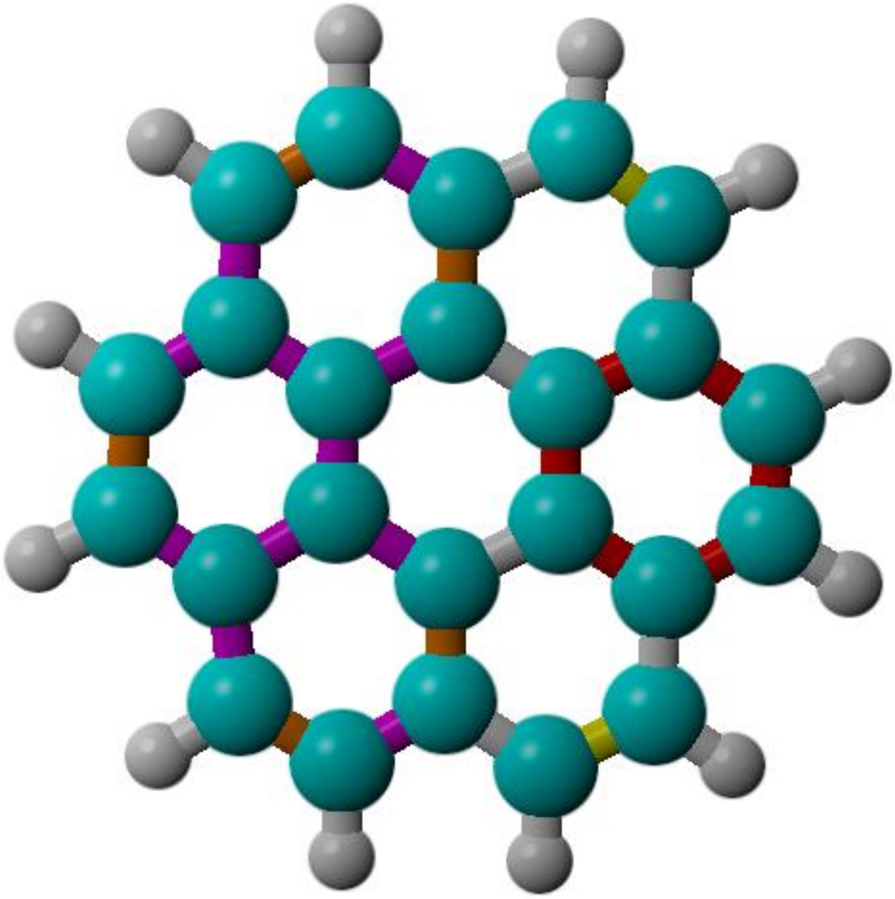

Figure S2-1. Coronene molecule built in YASARA Structure. Atoms: cyan, carbon; gray, hydrogen. Bonds: gray, bond of order 1; magenta, bond of order 1.33; red, bond of order 1.5; orange, bond of order 1.67 yellow, bond of order 2.

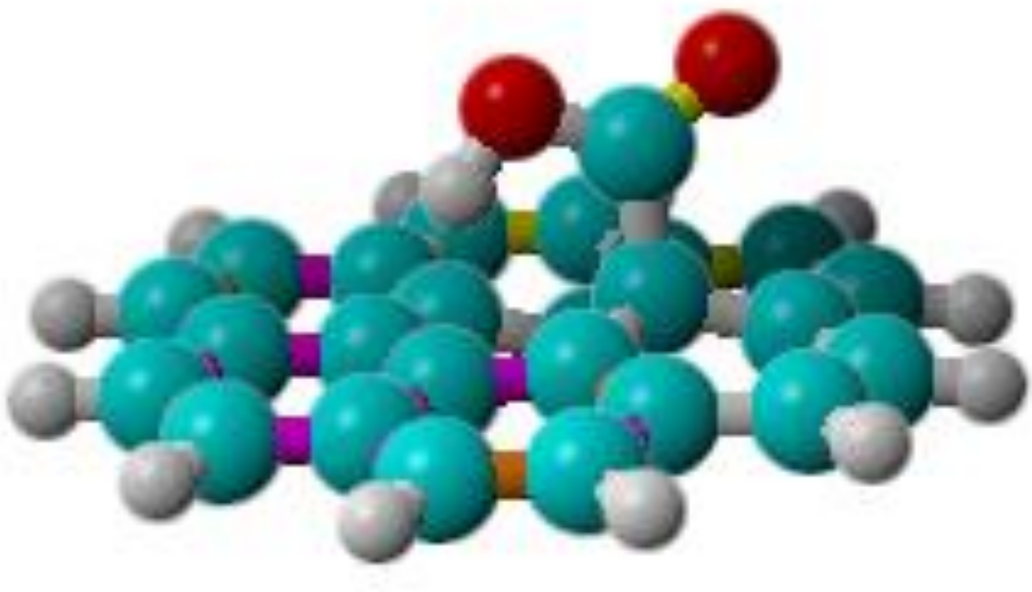

Figure S2-2. Coronene molecule carboxylated at the central position. Atoms: cyan, carbon; gray, hydrogen; red, oxygen. Bonds: gray, bond of order 1; magenta, bond of order 1.33; red, bond of order 1.5; orange, bond of order 1.67 yellow, bond of order 2.

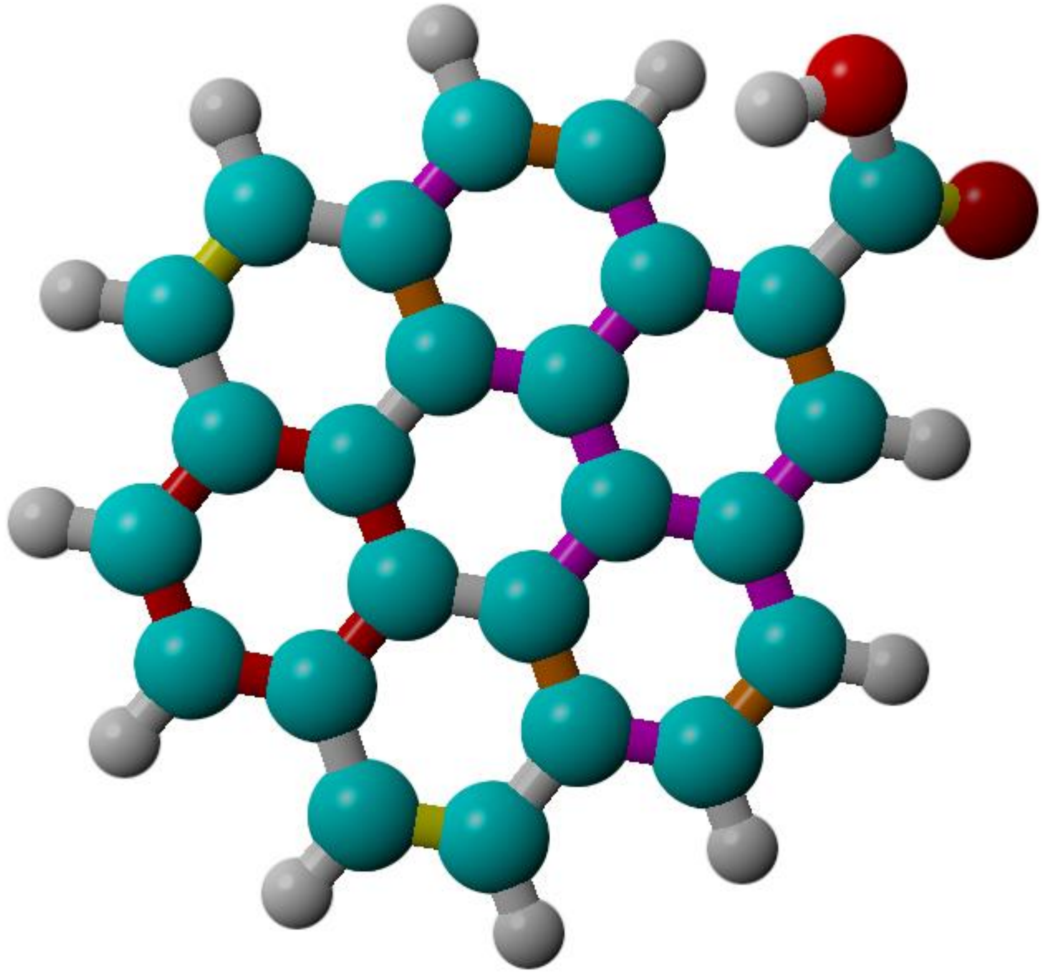

Figure S2-3. Coronene molecule carboxylated at the edge position. Atoms: cyan, carbon; gray, hydrogen; red, oxygen. Bonds: gray, bond of order 1; magenta, bond of order 1.33; red, bond of order 1.5; orange, bond of order 1.67 yellow, bond of order 2.

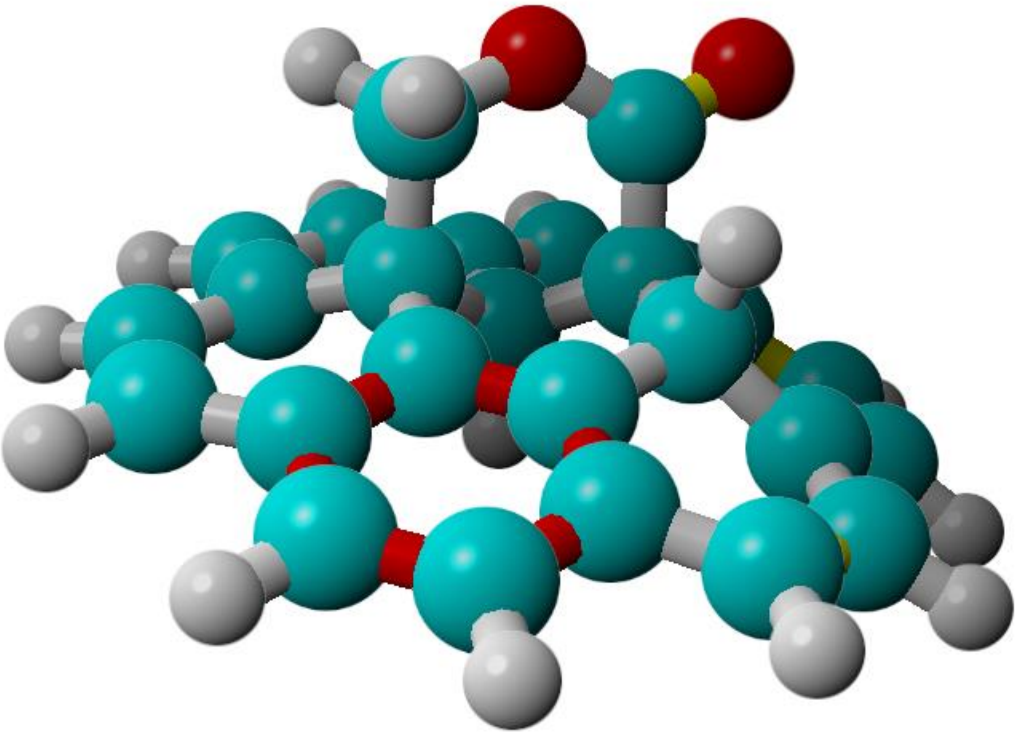

Figure S2-4. Coronene molecule with lactone group at the central position. Atoms: cyan, carbon; gray, hydrogen; red, oxygen. Bonds: gray, bond of order 1; magenta, bond of order 1.33; red, bond of order 1.5; orange, bond of order 1.67 yellow, bond of order 2.

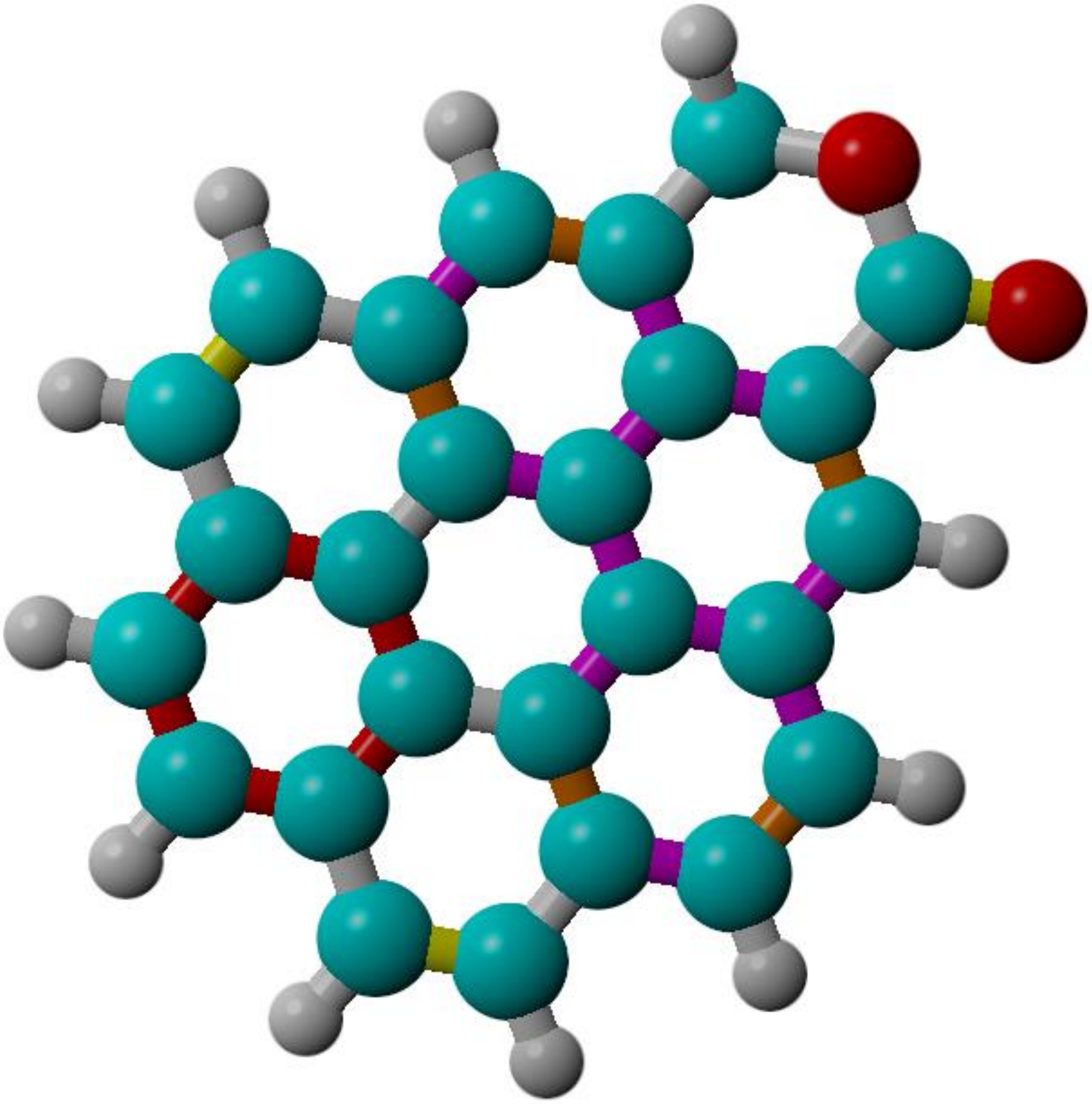

Figure S2-5. Coronene molecule with lactone group at the edge position. Atoms: cyan, carbon; gray, hydrogen; red, oxygen. Bonds: gray, bond of order 1; magenta, bond of order 1.33; red, bond of order 1.5; orange, bond of order 1.67 yellow, bond of order 2.

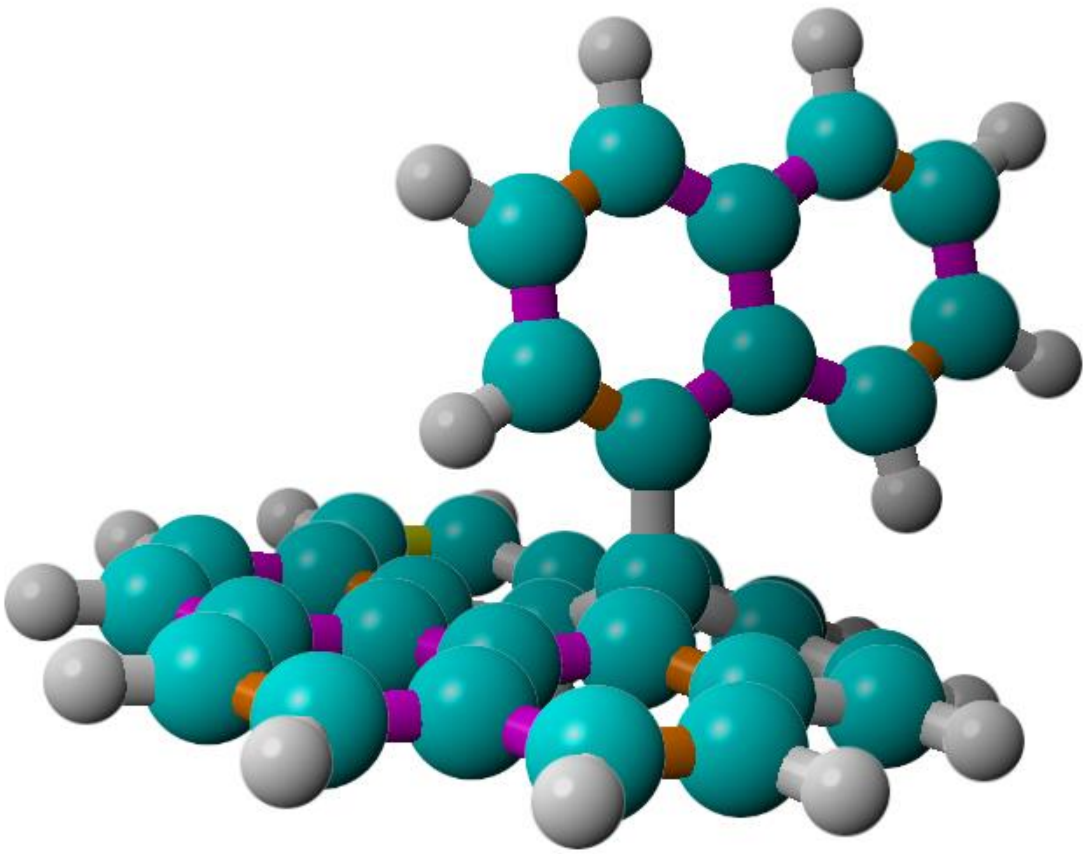

Figure S2-6. Coronene molecule with naphthyl group at the central position. Atoms: cyan, carbon; gray, hydrogen. Bonds: gray, bond of order 1; magenta, bond of order 1.33; red, bond of order 1.5; orange, bond of order 1.67 yellow, bond of order 2.

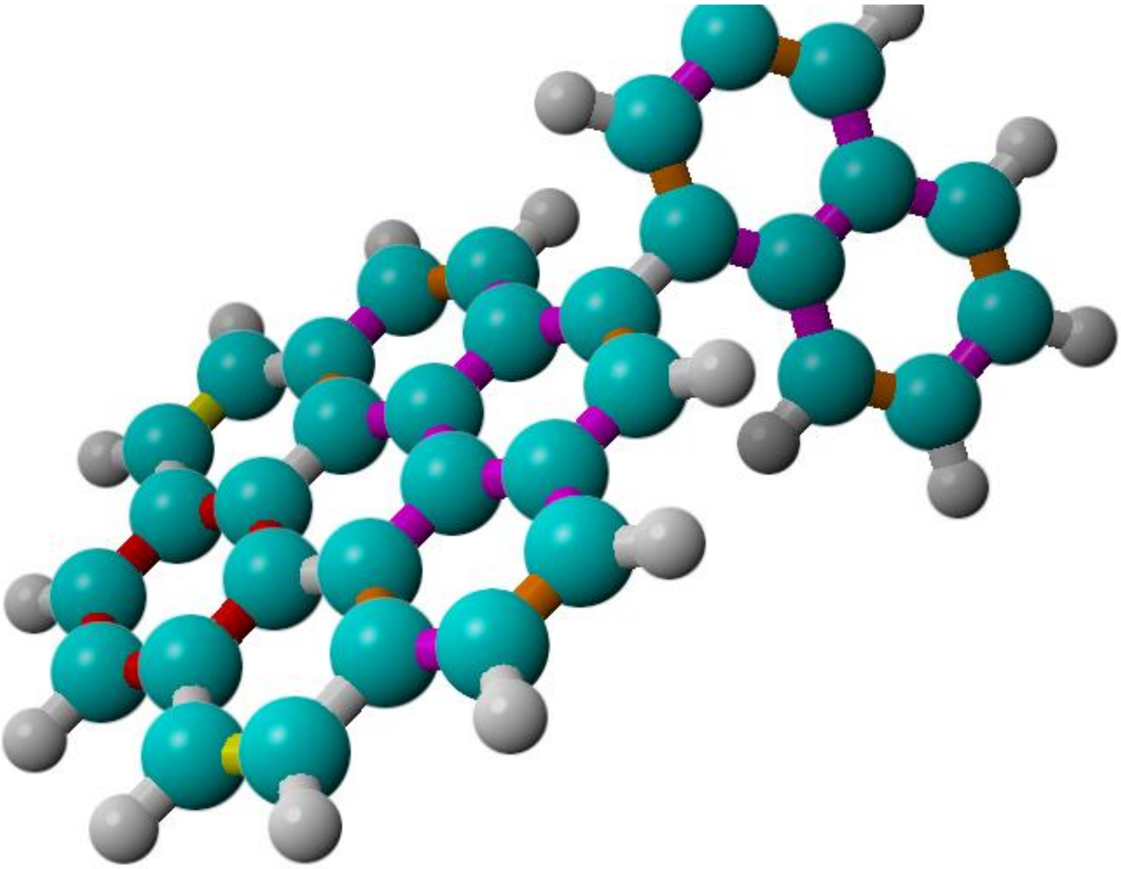

Figure S2-7. Coronene molecule with naphthyl group at the edge position. Atoms: cyan, carbon; gray, hydrogen. Bonds: gray, bond of order 1; magenta, bond of order 1.33; red, bond of order 1.5; orange, bond of order 1.67 yellow, bond of order 2.

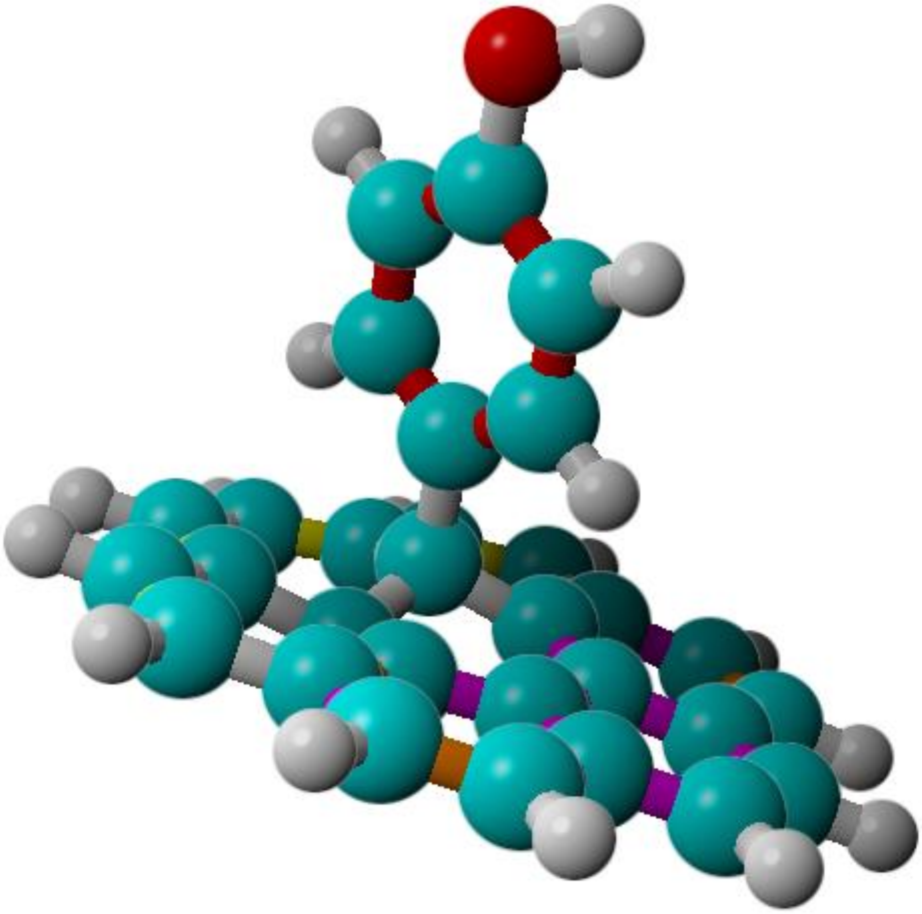

Figure S2-8. Coronene molecule with phenol group at the central position. Atoms: cyan, carbon; gray, hydrogen; red, oxygen. Bonds: gray, bond of order 1; magenta, bond of order 1.33; red, bond of order 1.5; orange, bond of order 1.67 yellow, bond of order 2.

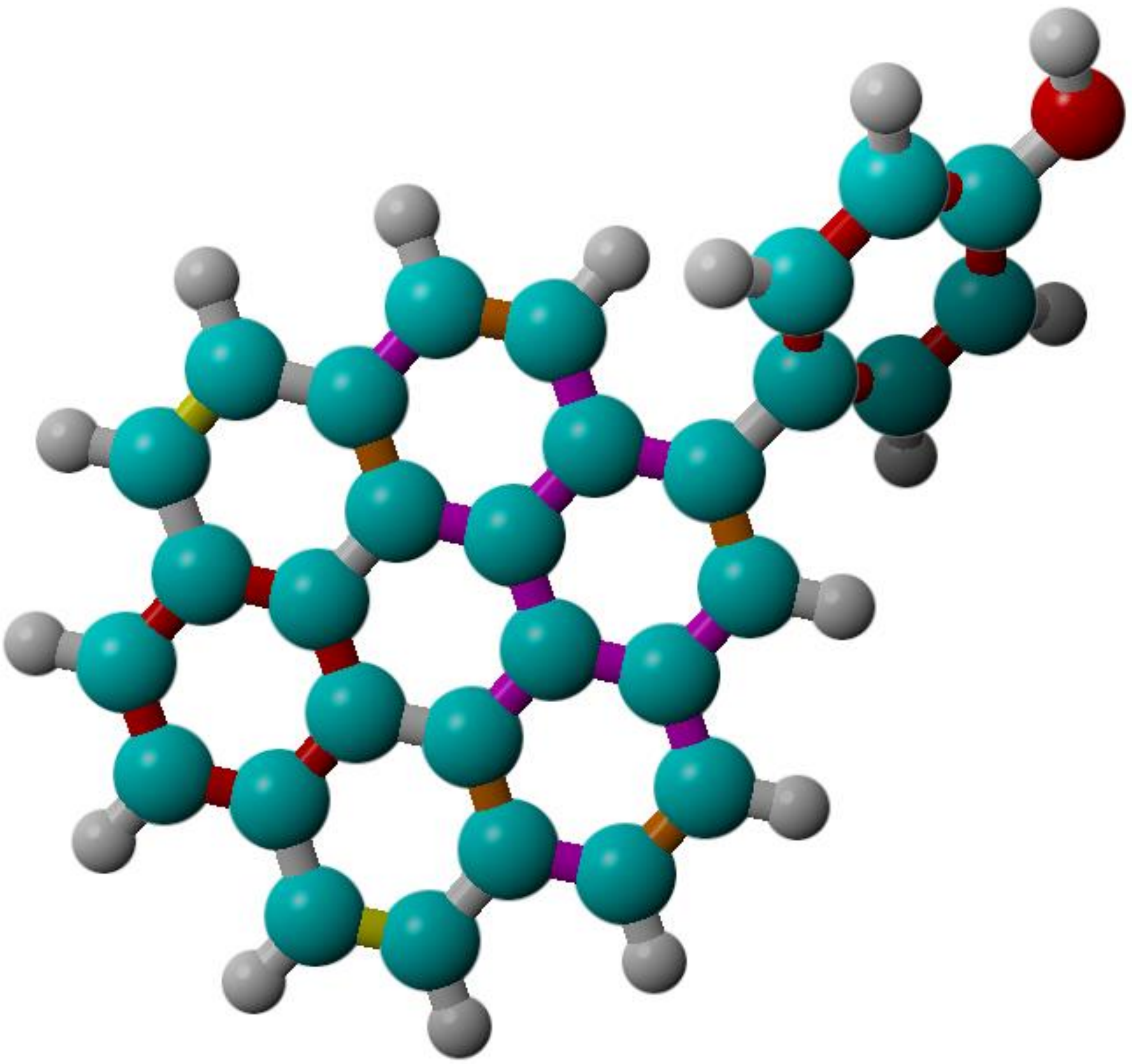

Figure S2-9. Coronene molecule with phenol group at the edge central position. Atoms: cyan, carbon; gray, hydrogen; red, oxygen. Bonds: gray, bond of order 1; magenta, bond of order 1.33; red, bond of order 1.5; orange, bond of order 1.67 yellow, bond of order 2.

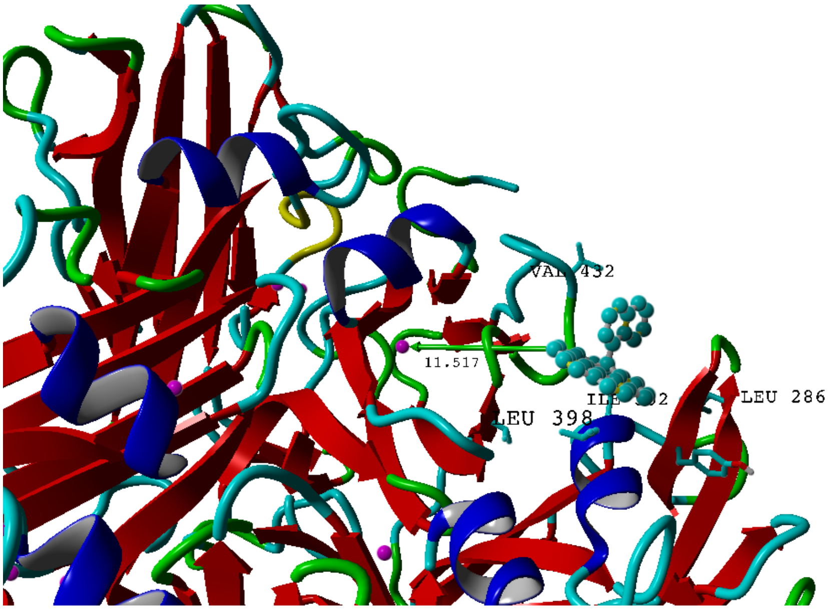

Figure S2-10. Binding of coronene with central naphthyl substitution to Ac-875 laccase. Surrounding hydrophobic amino acids are labeled; green arrow shows the shortest distance to the T1 copper atom (11.517 Å)

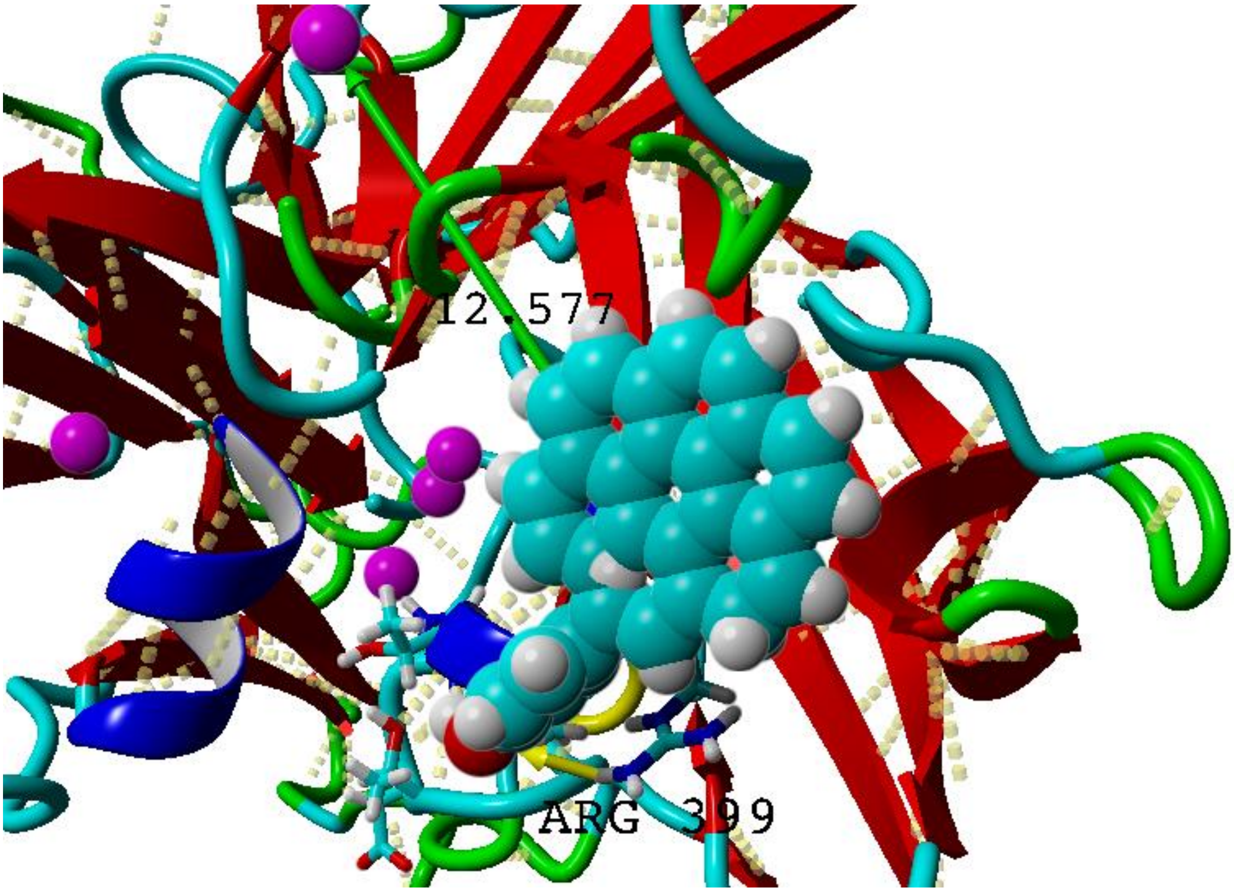

Figure S2-11. Binding of coronene with the edge phenol group to Ac-875 laccase. Hydrogen bonding network is shown as dashed lines; possible hydrogen bond between phenolic group and Arg399 is shown as solid yellow arrow; green arrow shows the shortest distance to the T1 copper atom (12.577 Å)
